# Pseudo-Luciferase Activity of the SARS-CoV-2 Spike Protein for *Cypridina* Luciferin

**DOI:** 10.1021/acscentsci.3c00887

**Published:** 2024-01-17

**Authors:** Ryo Nishihara, Hisham M. Dokainish, Yoshiki Kihara, Hiroki Ashiba, Yuji Sugita, Ryoji Kurita

**Affiliations:** † National Institute of Advanced Industrial Science and Technology (AIST), 1-1-1 Higashi, Tsukuba, Ibaraki 305-8566, Japan; ‡ Japan Science and Technology Agency (JST), PREST, 4-1-8, Honcho, Kawaguchi, Saitama 332-0012, Japan; § Faculty of Pharmaceutical Sciences, Hokkaido University, Nishi 6 Kita12 Kita-ku, Sapporo 060-0812, Japan; ¶ Theoretical Molecular Science Laboratory, RIKEN Cluster for Pioneering Research, 2-1 Hirosawa, Wako, Saitama 351-0198, Japan; # Faculty of Pure and Applied Sciences, University of Tsukuba, 1-1-1 Tennodai, Tsukuba, Ibaraki 305-8573, Japan; ∥ Laboratory for Biomolecular Function Simulation, RIKEN Center for Biosystems Dynamics Research, 6-7-1 Minatojima-minamimachi, Chuo-ku, Kobe, Hyogo 650-0047, Japan; ⊥ Computational Biophysics Research Team, RIKEN Center for Computational Science, 6-7-1 Minatojima-minamimachi, Chuo-ku, Kobe, Hyogo 650-0047, Japan

## Abstract

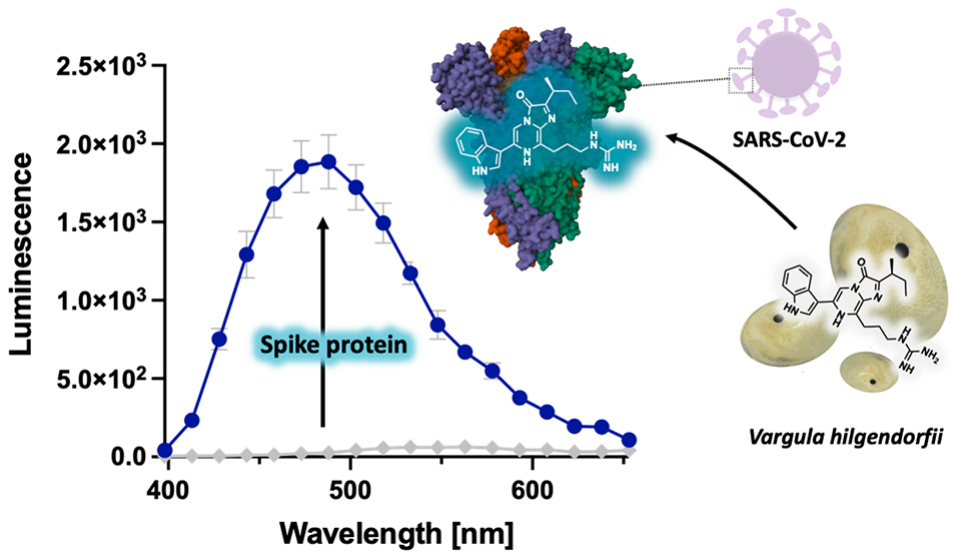

Enzymatic reactions that involve a luminescent substrate (luciferin) and enzyme (luciferase) from luminous organisms enable a luminescence detection of target proteins and cells with high specificity, albeit that conventional assay design requires a prelabeling of target molecules with luciferase. Here, we report a luciferase-independent luminescence assay in which the target protein directly catalyzes the oxidative luminescence reaction of luciferin. The SARS-CoV-2 antigen (spike) protein catalyzes the light emission of *Cypridina* luciferin, whereas no such catalytic function was observed for salivary proteins. This selective luminescence reaction is due to the enzymatic recognition of the 3-(1-guanidino)propyl group in luciferin at the interfaces between the units of the spike protein, allowing a specific detection of the spike protein in human saliva without sample pretreatment. This method offers a novel platform to detect virus antigens simply and rapidly without genetic manipulation or antibodies.

## Abstract 2

The spike protein, which decorates the SARS-CoV-2, catalyzes the oxidative luminescence reaction of *Cypridina* luciferin, a substrate for *Cypridina* luciferase.

## Introduction

Light-emitting enzymatic reactions are widespread in nature and are widely used in biotechnology.^1,2^ The luminescence associated with the oxidative reaction of the substrate luciferin by the corresponding enzyme luciferase is the basis of methods for the bioluminescence (BL) detection of gene expression, proteins, and cells.^1^ The luciferin–luciferase pair varies among luminescent organisms, and its enzymatic reaction proceeds specifically, allowing the selective detection of luciferase-conjugated target biomolecules in vitro and in vivo. However, from a technical standpoint, general BL-based assays require prior genetic modification of the target biomolecule via the incorporation of luciferase genes.

**Scheme 1:**
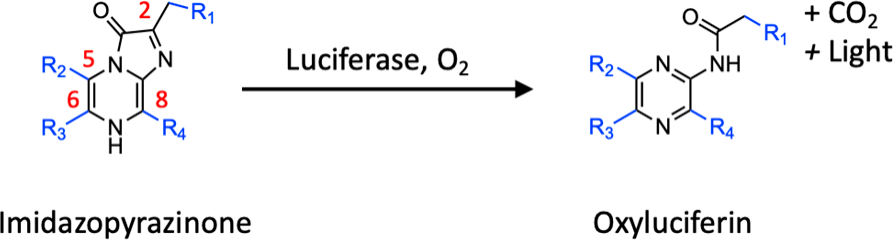
Enzymatic Oxidate Reaction of Imidazopyrazinone into Oxyluciferin with Concomitant Emission of Light The C-2, C-6, and C-8 substituents (R_1_–R_4_) of the imidazopyrazinone ring differ among luciferases of different luminous marine organisms.

**Figure 1:**
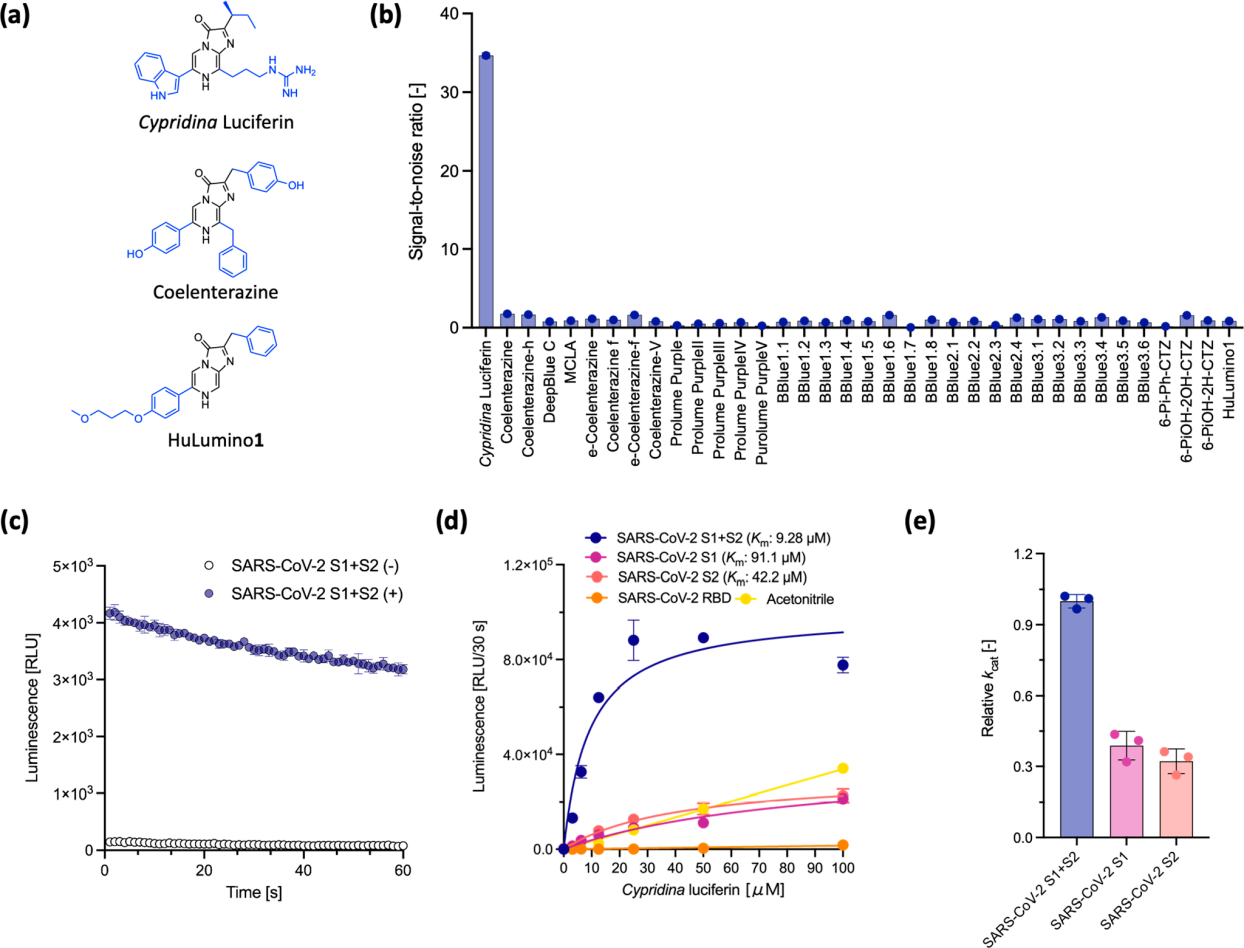
(a) Chemical structures of *Cypridina* luciferin, coelenterazine, and HuLumino**1**. (b) Luminescence response from monomer S protein (72 nM) treated with imidazopyrazinone-type luciferins (20 μM). The signal-to-noise ratio indicates the ratio of the total luminescence of the luciferin/S protein pair relative to that of luciferin only. (c) Luminescence decay of *Cypridina* luciferin (20 μM) in the presence of monomeric S protein (72 nM). (d) Dose-dependent luminescence intensities: *Cypridina* luciferin (0–100 μM) in the presence of S protein (72 nM) or acetonitrile. (e) The relative *k*_cat_: *k*_cat_ value compared to that of the *Cypridina* luciferin/full-length S protein (72 nM) pair was calculated from the *V*_max_ values, determined from (d). Error bars represent the standard deviation of the three measurements.

At present, structural analysis of luciferin is more advanced than that of luciferase, and it is known that the chemical structure of luciferin is diverse among the various species of luminous organisms. In particular, imidazopyrazinone-type (IPT) luciferin is widespread in luminous marine organisms (Scheme 1). The diversity of the BL systems in different marine organisms originates from the chemical structure of the side-chain of IPT luciferins in addition to luciferase evolution.^2^ For example, *Cypridina* luciferin and coelenterazine (CTZ) are specifically catalyzed by *Cypridina* luciferase (Cluc) derived from the ostracods of the family Cypridinidae such as *Vargula* (*Cypridina*) *hilgendorfii* and *Renilla* luciferase derived from sea pansy *Renillareniformis*, respectively (Figure 1a).^1^

Although a luciferin typically emits light only in the presence of the corresponding luciferase, IPT luciferin often exhibits nonspecific luminescence reactions toward nonluciferase proteins or other biomolecules, as observed in both cellular- and animal-imaging studies.^3^ To date, some reports have revealed that nonluminescent proteins, such as serum proteins including human plasma alpha 1-acid glycoprotein,^4^ bovine serum albumin,^3^ and insulin,^5^ can catalyze the nonspecific luminescence oxidative reaction of natural IPT luciferin, even though they have not been assigned an EC number and are not recognized as enzymes.

We have recently demonstrated that CTZ derivative HuLumino**1**, which is an IPT luciferin with a side-chain chemical structure that is different from that of CTZ, is selectively recognized and enzymatically oxidized to exhibit light by one of several hydrophobic pockets (drug-binding site 2) in human serum albumin (HSA).^6,7^ However, this luminescence was not observed in bovine serum albumin, which has 75.6% homology to HSA, or other proteins. Interestingly, HuLumino**1** reacts only with HSA, even in human serum containing various proteins, and has the capability to quantitatively detect HSA with the same precision as an enzyme-linked immunosorbent assay (ELISA) in only 1 min. This discovery suggested that protein assays based on the “pseudoluciferase activity” of the targeted protein itself may potentially serve as a novel platform for protein analysis and alternative luminescence assays that do not rely on luciferase derived from luminous organisms. We here refer to this specific luminescence reaction by nonluciferase biomolecules as “biomolecule-catalyzing chemiluminescence (BCL)”. However, in the context of BCL-based protein assays, further identification of types of proteins with pseudoluciferase activity and rational design of luciferins that can be selectively catalyzed by a nonluminescent wild protein of interest are required to extend the possible application of BCL systems to proteins other than serum proteins.

In 2019, the global coronavirus pandemic (COVID-19) caused by SARS-CoV-2^8^ led to the rapid development of treatment and diagnostics that target viral RNA and antigens.^9^ Among these target molecules, the spike (S) protein, an antigen decorated on the surface of the virus particles, is a transmembrane trimeric glycoprotein and plays an important role to assist the virus to enter human cells by using the receptor-binding domain (RBD).^10,11^ Hence, S proteins have been characterized using structural analysis and computational simulations as having multiple “hydrophobic pockets” that can bind a variety of ligands such as linoleic acid, which binds to the pockets on the interfaces between RBDs, thus reducing the ability of viral infection.^12,13^

Moreover, some studies have suggested that the luminescence reaction of IPT luciferin requires a hydrophobic pocket as an oxidative reaction field.^4,6,14^ Therefore, we hypothesized that specific luminescence detection of the S protein could be achieved by selecting a luciferin that specifically fits into the hydrophobic pockets of the S protein.

In this study, we report that the S protein of SARS-CoV-2 (wild type) has pseudoluciferase activity for *Cypridina* luciferin and clarify the correlation between its chemical structure of luciferin and luminescence activity using luciferin analogues. The development of a BCL-based assay system for the selective and rapid (within 1 min) detection of S protein in human saliva without sample pretreatment shows promising potential to complement centralized reverse-transcription polymerase chain reaction (RT-PCR)-based testing, which requires dedicated clinical facilities, trained personnel, and long times for diagnosis.^9^

## Results and Discussion

### Luciferin to Reveal the Pseudoluciferase Activity in the Monomeric S Protein

We first screened IPT luciferins to generate light emission with the full-length monomer of the SARS-CoV-2 S protein. As IPT luciferin requires no cofactors other than an oxygen molecule^1^ (Scheme 1), the luminescence assay was employed to read luminescence signals after simply mixing luciferin and the S protein. Here, 36 IPT luciferins, including two native luciferins (CTZ and *Cypridina* luciferin) and 34 reported CTZ analogues,^3^ in which the substituents at the C-2, C-5, C-6, and C-8 positions of the imidazopyrazinone-ring were varied relative to those of native luciferin (Figure 1a and Figure S1), were mixed with S protein. In the assay, the monomeric S protein showed light emission with only *Cypridina* luciferin (signal-to-noise ratio (S/N) of 34.7) but not with other luciferins (S/N near or below 1) (Figure 1b). The suitable orthogonal pair of *Cypridina* luciferin/monomeric S protein exhibited flash-type kinetics, which are a common characteristic in BL systems using IPT luciferin,^15^ with an approximately 23% decrease in luminescence intensity over 1 min (Figure 1c). These results indicate that IPT luciferin containing *sec*-2-butyl, 3-indolyl, and 3-(1-guanidino)propyl groups at C-2, C-6, and C-8 of the imidazopyrazinone ring, respectively, reveals the pseudoluciferase activity of the SARS-CoV-2 S protein.

We then identified the possible subunits of the monomeric S protein in which the luminescence reaction of *Cypridina* luciferin might occur using the three fragments (S1, S2, and RBD) of the S protein (Figure S2a).^10^ Their kinetic profiles were compared, and the apparent *K*_m_ value as well as the relative *k*_cat_ value (determined based on the *V*_max_ value) were calculated from the Michaelis–Menten equation fitting curves plotted using the initial luminescence intensity for the first 30 seconds (Figure 1d,e). For the S1 + S2 full-length protein, the *K*_m_ value was calculated to be 9.28 μM (Figure 1d and Table S1). In contrast, the *K*_m_ values for the S1 and S2 units were 91.1 and 42.2 μM, respectively, and no luminescent signal was observed for RBD and its variants (Y453F and N501Y) (Figures 1d, S2b and Table S1). Furthermore, the S1+S2 full-length monomeric S protein showed a higher catalytic efficiency for *Cypridina* luciferin than the fragment proteins, increasing its relative *k*_cat_ by more than a factor of 2.6 (Figure 1e and Table S1). This result suggests that the reaction sites formed when the units combine, such as the interfaces between S1 and S2, may contribute to the luciferin luminescence rather than the individual units themselves.

Considering the low *K*_m_ values of the natural Cluc system (0.52 μM),^1^ the *K*_m_ values of fragment proteins are 2 orders of magnitude higher than that of the natural BL system (>42 μM) (Figure 1d and Table S1). Comparing the chemiluminescence (CL) system in which *Cypridina* luciferin exhibits luciferase-independent luminescence in polar aprotic solvents such as acetonitrile^16^ with the S-fragment luminescence systems, we observed that the fitting curves obtained with the fragments differ slightly from the luciferin-concentration-dependent linear response obtained with acetonitrile, albeit both luminescence levels are similar (Figure 1d). Consequently, this fragment-protein-driven luminescence should be categorized into the BCL based on its luminescence intensities. We then examined whether the BCL reaction of the *Cypridina* luciferin/S1-fragment protein pair was specific to SARS-CoV-2. SARS-CoV (severe acute respiratory syndrome coronavirus) and MERS-CoV (Middle East respiratory syndrome coronavirus) have high genome similarity to SARS-CoV-2^8,17^ and are known to use their own S protein to access host cells.

In a comparative test, the relative *k*_cat_ values of the S1 proteins were higher for SARS-CoV-2 and SARS-CoV (in that order), while MERS-CoV did not plateau, not even at a luciferin concentration of 100 μM, and its *V*_max_ value could not be calculated as well as with acetonitrile (Figure S3 and Table S2). Since SARS-CoV-2 has 79% gene sequence homology with SARS-CoV and 50% with MERS-CoV, *Cypridina* luciferin is likely to emit CL catalyzed by a structure unique to SARS-associated coronaviruses at least. In contrast, the S1 + S2 full-length monomeric S protein showed a much higher affinity (*K*_m_: 9.28 μM) and catalytic efficiency for *Cypridina* luciferin than the fragment proteins (Figure 1d,e), although its luminescence intensity is comparable to that of CL obtained with acetonitrile (Figure 1d). The luminescence quantum yield, which is an index of brightness, of *Cypridina* luciferin in a CL system with acetonitrile including sodium acetate buffer has been reported to be only 6.6% of the Cluc BL system.^1^ Certainly, the monomeric S protein has pseudoluciferase activity to catalyze the luminescence reaction of *Cypridina* luciferin while having a *K*_m_ value similar to Cluc, albeit that it is probably more appropriate to refer to this phenomenon as BCL rather than BL.

### BCL Reaction in the Trimeric S Protein

**Figure 2:**
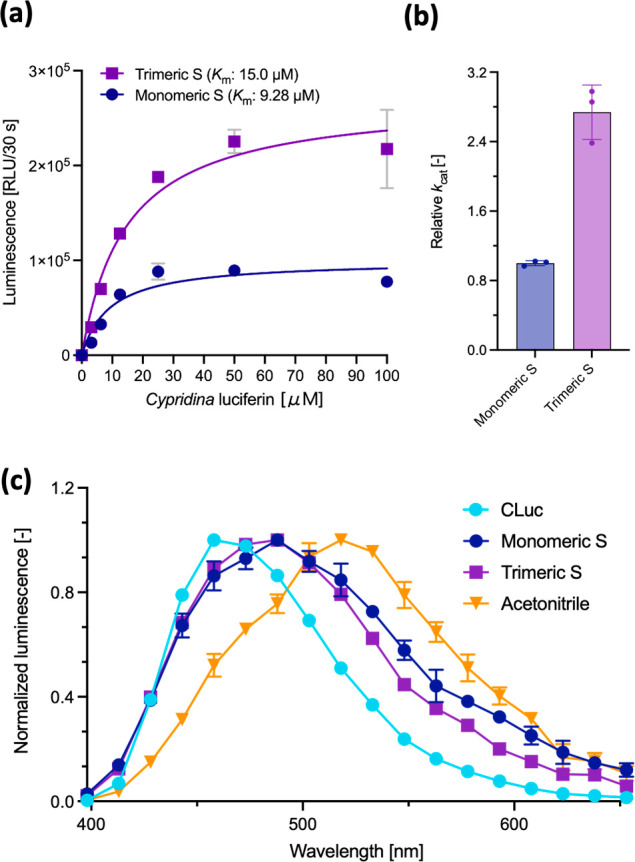
(a) Dose-dependent luminescence intensities: *Cypridina* luciferin (0–100 μM) in the presence of SARS-CoV-2 S proteins (72 nM). (b) The relative *k*_cat_: *k*_cat_ value compared to the *Cypridina* luciferin/monomeric S protein (72 nM) pair was calculated from the *V*_max_ values, determined from (a). (c) Luminescence spectra of *Cypridina* luciferin in the presence of proteins or acetonitrile. Error bars represent the standard deviation of three individual measurements.

The S protein is folded in a trimeric form on the surface of the SARS-CoV-2 virus particle.^10^ Given that the assays with the monomeric S protein showed that the interfaces between the units potentially play a role in the molecular recognition of luciferin (Figure 1d), here the effect of the interface of the S protein on the luminescence reaction was addressed by using the trimeric S protein. At all luciferin concentrations, the trimeric S protein exhibited a luminescence intensity approximately twice as high as that of the monomeric one (Figure 2a). Based on the Michaelis–Menten equation fitting curves in Figure 2a, we concluded that *Cypridina* luciferin has higher affinity for the monomeric S protein than the trimeric one (*K*_m_ values of 9.28 μM for the monomer and 15.0 μM for the trimer), whereas the relative *k*_cat_ value for the trimeric S protein is by a factor of approximately 2.7 higher than that for the monomeric S one (Figure 2b and Table S3). In the trimeric S protein, the interfaces between the monomers and/or subunits are reported to be potential binding sites for the ligands.^18,19^ There are more interfaces between domains in the trimer than in the monomer, which may require a larger amount of luciferin to reach the steady state, resulting in a relatively high reaction efficiency.

The BCL reaction could also be observed in the trimeric S protein of the SARS-CoV-2 variant (Omicron, BA.2.12.1), catalyzing the luminescence reaction of *Cypridina* luciferin (Figure S4). This is due to the overall organization of the trimeric S protein, which is conserved between wild-type and omicron variant.^20^ As a result of the amino-acid mutations, the catalytic efficiency of the omicron variant was reduced to half of that of the wild type, with a relatively high *K*_m_ value of 49.6 μM (Figure S4 and Table S4). Hence, amino-acid mutations have a significant effect on kinetic profiles, but other mutant S proteins, in which the structure of the trimeric S protein (wild type) is conserved, may also exhibit pseudoluciferase activity.

In the BL reaction, the emission spectrum is determined by the luminescent oxyluciferin species derived from chemical (proton tautomeric) equilibria, which reflect the microenvironment (e.g., polarity, basicity, hydrophobicity, and the geometry of the reaction site) around the luciferin in the luciferase molecule.^1^*Cypridina* luciferin exhibits an emission with maximum wavelength (λ_max_) values of 458, 488, and 518 nm in the presence of Cluc, S proteins, and acetonitrile, respectively (Figure 2c), indicating that the microenvironment of the reaction site differs in each case. Moreover, the full width half-maximum (fwhm) values, i.e., the width of the spectrum where the emission is half the value of the maximum, which represents an index of the number of light-emitting species in the excited state,^21^ were 87, 122, 107, and 118 nm for Cluc, monomeric S protein, trimeric S protein, and acetonitrile, respectively. The broader fwhm for the S proteins is due to the presence of more light-emitting species than that in the case of Cluc. Although a structural analysis of Cluc has not yet been achieved, for other IPT luciferins, it has been proposed that there is one enzymatic reaction pocket per luciferase molecule.^22^ This wide fwhm value may suggest that, unlike in the Cluc system, the S protein features multiple luciferin-binding pockets that make the excited-state light emitters more complex.

### Structure–Activity Relationship between Luciferin Analogues and the SARS-CoV-2 Spike Protein

**Figure 3:**
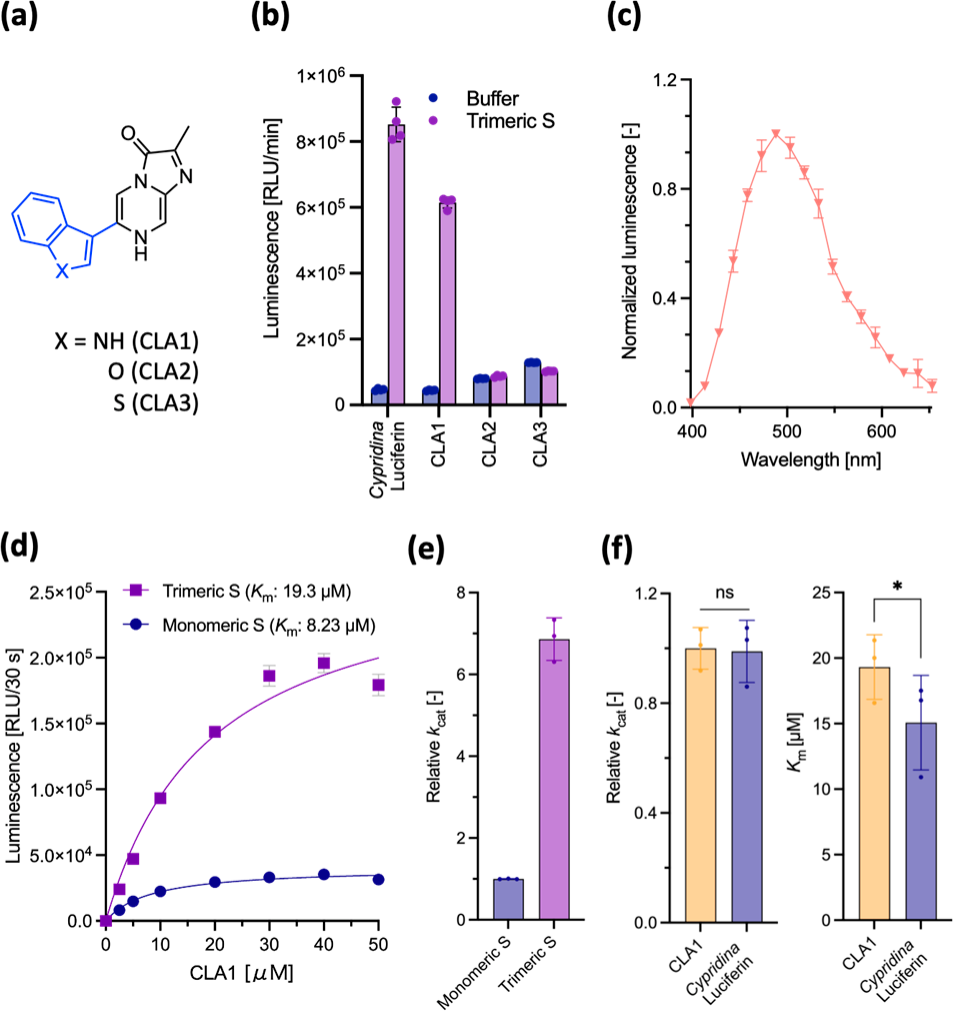
(a) Chemical structures of the CLAs used in this study. (b) Luminescence response from trimeric S protein (72 nM) treated with *Cypridina* luciferin and CLA1–3 (20 μM). (c) Normalized luminescence spectrum of CLA1 in the presence of the trimeric S protein. (d) Dose-dependent luminescence intensities: CLA1 (0–50 μM) in the presence of SARS-CoV-2 S proteins (72 nM). (e) Relative *k*_cat_: *k*_cat_ values compared to the CLA1/monomeric S protein (72 nM) pair were calculated from the *V*_max_ values, determined from (d). (f) Relative *k*_cat_ and *K*_m_ between CLA1 and *Cypridina* luciferin in the presence of trimeric S protein (72 nM); *P < 0.03 (*t*-test). Error bars represent the standard deviation of three independent measurements.

To obtain insight into the enzymatic luminescence reaction of *Cypridina* luciferin and the SARS-CoV-2 S protein, we assessed the structure–activity relationship between *Cypridina* luciferin analogues (CLAs) and the S protein. The 3-indolyl and 3-(1-guanidino)propyl groups are characteristic functional groups of *Cypridina* luciferin that have not been found in other elucidated luciferins that occur naturally. To access the effect of these functional groups on the enzymatic luminescence activity of the S protein, three types of CLAs were prepared by replacing the NH moiety of the 3-indolyl group at the C-6 position of the imidazopyrazinone ring with another heteroatom and eliminating the 3-(1-guanidino)propyl group at the C-8 position using reported synthetic procedures^16^ (Figure 3a).

First, the effect of the C-6 substituent on the luminescence intensity was examined. Luminescence assays using trimeric S protein revealed that CLA1, which has an indolyl group, exhibited approximately 70% of the luminescence intensity of *Cypridina* luciferin at the same emission wavelength as *Cypridina* luciferin, indicating that even a luciferin without the 3-(1-guanidino)propyl group can react with the S protein and emit light (Figure 3b,c). In contrast, luciferin with a 3-benzofuranyl group (CLA2) or 3-benzothienyl group (CLA3) showed no response to the S protein (Figure 3b). These results suggest that the 3-indolyl group plays an important role in the reaction with the S protein (Figure 3a,b). CLA1 itself has high luminescence ability, as it has been reported to have a higher CL quantum yield than CLA2 and CLA3.^16^ Therefore, the luminescence intensity with the S protein is expected to correspond to the C-6-substituent-related inherent luminescence properties of the luciferin.

We then envisioned that the 3-(1-guanidino)propyl group at the C-8 position of the *Cypridina* luciferin could play a major role in the binding affinity. To assess this hypothesis, the luminescence kinetic profiles of CLA1 were compared to those of *Cypridina* luciferin. Based on the results of a dose–response luminescence assay (Figure 3d), CLA1 also exhibits a higher binding affinity toward the monomer than toward the trimer, although the luminescence intensity and catalytic efficiency are higher for the trimer, as was observed with *Cypridina* luciferin (Figures 3d,e as well as Table S5). A comparison of the kinetic profiles obtained with the trimeric S protein revealed that the relative *k*_cat_ of *Cypridina* luciferin is the same as that of CLA1 (Figure 3f as well as Tables S3 and S5), albeit that the binding affinity of *Cypridina* luciferin is higher (Figure 3f; the *K*_m_ values of *Cypridina* luciferin and CLA1 are 15.0 and 19.3 μM, respectively).

Based on these kinetic profiles, it can be concluded that the 3-indolyl group of the C-6 substituent is dominant in determining the luminescence intensity and that the 3-(1-guanidino)propyl group at the C-8 substituent improves the binding affinity to the S protein in the *Cypridina* luciferin/S protein luminescence system. The natural Cluc/*Cypridina* luciferin pair has the highest BL quantum yield (30%) among all BL systems involving IPT luciferin,^1^ and the reaction is extremely specific, with no cross-reactivity with the natural substrate coelenterazine or other IPT luciferins (Figure S5). In the Cluc system, the contribution of the individual substituents of *Cypridina* luciferin to the Cluc BL has not been elucidated so far, because the luminescence intensity is significantly reduced when any of the substituents are eliminated.^23^ In contrast, in the S protein luminescence system, the functions of the substituents of *Cypridina* luciferin were clearly determined. These findings are expected to contribute greatly to the unknown reaction mechanism of Cluc BL systems.

### Computational Calculation of Potential Binding Site and Binding Affinity of Luciferin with the SARS-CoV-2 S Protein

**Figure 4:**
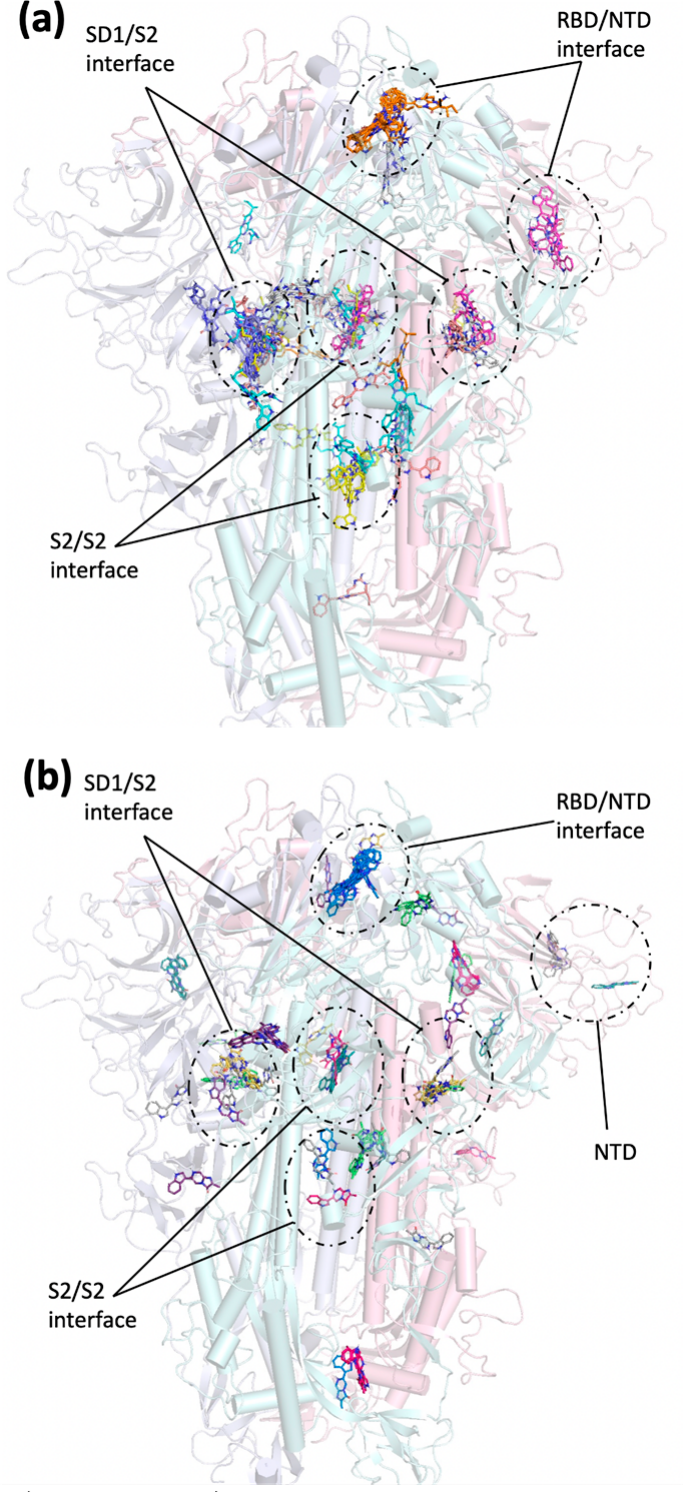
Docking simulation of the trimeric S protein based on MD simulations clusters center with (a) *Cypridina* luciferin and (b) CLA1 as predicted by Autodock Vina. The top-ten-ranked poses from all seven docked conformations are indicated.

The high binding affinity of *Cypridina* luciferin to the S protein is due to the 3-(1-guanidino)propyl group at the C-8 position, and the role of this substituent in binding the S protein was investigated using computational simulations with Autodock Vina.^24^ First, to predict the luminescence reaction site, a blind docking simulation was performed in which the whole trimeric S protein was selected as a receptor. The trimeric S protein is known to form several conformational states because of its inherent flexibility. In the docking-simulation test with luciferins, we used seven clustered S protein conformations based on the structural organization of RBD, which we have already reported elsewhere.^18^ Given that the trimeric SARS-CoV-2 S protein exhibits a “down” conformation of the RBDs in its prefusion conformation to escape from immune response,^25^ we tested structures that represent different down conformations of the RBDs, including symmetric down conformations (D1_Sym_, D2_Sym_), asymmetric down conformations in which one or more RBDs adopt different hinge/twist angles (D1_Asym_, D2_Asym_), and Intermediate 1 conformations (Ia, Ib, Ic), in which the RBDs show a slight increase in hinge angle.^18^ An analysis of the ten top-ranked poses of *Cypridina* luciferin with all seven docked conformations predicted multiple luciferin-binding pockets at the interfaces of RBD/N-terminal domain (NTD), RBD/RBD, S2/S2, and subdomain 1 (SD1)/S2 (Figure 4a). Among all predicted binding pockets, the SD1/S2 and S2/S2 interfaces were identified in all conformers (Figure 4a), while some of the observed pockets might be transient, as they occurred only in one S protein conformation, such as those at RBD/NTD and RBD/RBD. In particular, the RBD/RBD interface of the trimeric S protein has been identified by a cryo-EM study as the only ligand binding site that binds to linoleic acid,^12^ albeit that blocking this interface pocket with linoleic acid does not inhibit the luminescence (Figure S6), indicating that the RBD/RBD interface is not a luminescence reaction site for *Cypridina* luciferin.

The binding sites of CLA1 in the trimeric S protein almost overlap with those of *Cypridina* luciferin, but CLA1 tends to be less specific and more diverse than *Cypridina* luciferin due to its smaller molecular size, as observed from its additional binding to NTDs (Figure 4b). A comparison of the binding free-energy calculations revealed that *Cypridina* luciferin has a higher binding affinity than CLA1 in all conformations of the trimeric S protein except for I1c (Table S6: the average binding free energy of *Cypridina* luciferin and CLA1 for S protein are −9.278 and −8.697 kcal/mol, respectively). These results align with the experimental findings in which *Cypridina* luciferin showed better binding affinity in the trimeric S protein than CLA1. Moreover, refined docking simulations specifying interfaces (NTD/RBD, SD1/S2 and S2/S2/S2) as receptors, in which the grid spacing was reduced from 1 Å (blind docking) to 0.375 Å, also showed that *Cypridina* luciferin has a binding affinity higher than that of CLA1 for the trimeric S protein (Figure S7a) due to the formation of multiple hydrogen bonds and hydrophobic interactions between luciferin and the amino acids in an interface (Figure S7b).

In summary, detailed investigations of the luminescence characteristics and the simulation analysis of the trimeric S protein with the luciferins suggested that (i) the 3-indolyl group at the C-6 position of the imidazopyrazinone ring affects the luminescence intensity; that (ii) the 3-(1-guanidino)propyl group at the C-8 position is involved in the binding affinity; and that (iii) multiple binding, mainly in the interfaces between units, may make the species of oxyluciferin in the excitation states more complex. This results in the observed broad emission spectra and high *K*_m_ values (Figures 2c and 3c), although it cannot be determined at this point whether all of the binding sites are involved in the oxidative reaction of luciferin.

### SARS-CoV-2 S Protein Assay in Human Saliva

The viral genes and antigens from human saliva are biomarkers for COVID-19 diagnosis.^26^ The detection of viral RNA by RT-PCR is the current gold standard for the diagnosis of COVID-19, albeit that it requires relatively expensive equipment, trained technicians, and a long diagnosis time (up to 2 h).^9^ In contrast to viral RNA detection, antigen detection based on lateral flow assay (LFA) is simpler, faster, and less expensive, making it ideal for developing countries where RT-PCR is difficult to implement.^26^ The current most useful antigen biomarkers for COVID-19 diagnosis are the S protein and the nucleocapsid (N) protein. Lateral-flow immunoassays represent a common method for analyzing these antigens, in which the specimen is simply placed in the device, and SARS-CoV-2 S antigen can be detected at LODs in the μg/mL to ng/mL range without pretreatment, with a detection time of 16–30 min.^26^

**Figure 5:**
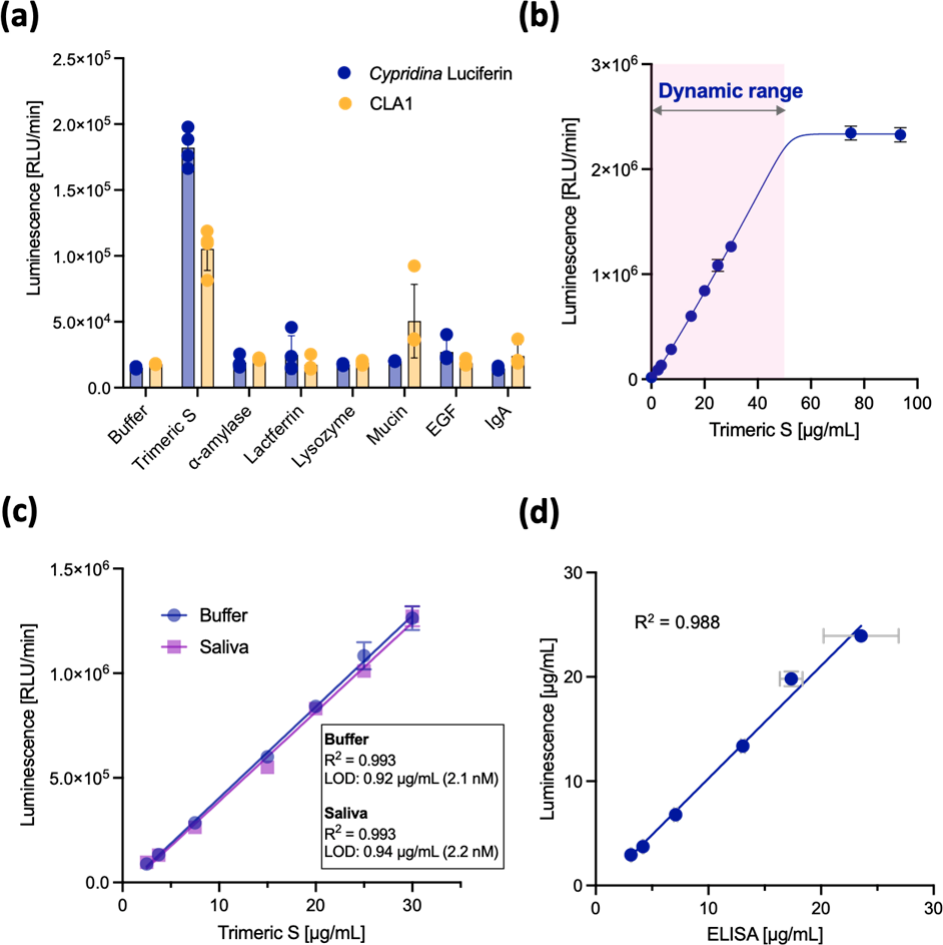
Luminescence intensity of *Cypridina* luciferin (20 μM) (a) in the presence of SARS-CoV-2 S protein or saliva proteins (10 μg/mL); (b) in buffer containing trimeric-S-protein concentrations of 0.5–93.6 μg/mL; (c) in buffer systems or saliva systems containing 0.5–30 μg/mL of the trimeric S protein. (d) Correlation between the measured concentrations of SARS-CoV-2 S protein using luminescence and ELISA. The markers and error bars represent the average and standard deviations of three independent measurements.

We investigated whether a BCL-based assay system using the pseudoluciferase activity of the S protein could detect the trimeric S protein in human saliva from a COVID-19 PCR-negative donor without sample pretreatment. First, we investigated the cross-reactivity of *Cypridina* luciferin and CLA1 with salivary proteins. Screening of *Cypridina* luciferin with six salivary proteins revealed that only the trimeric S protein led to a distinct luminescence enhancement; the other salivary proteins did not result in any light emission (Figure 5a). Interestingly, CLA1 showed a response to mucins in addition to that of the trimeric S protein (Figure 5a). This may be because CLA1, as a smaller molecule than *Cypridina* luciferin, has access to the potential oxidative reaction sites of mucins. As shown in Figure 5a, the 3-(1-guanidino) propyl group increases the reaction selectivity of luciferin for the S protein. This indicates that the substituent at the C-8 position of IPT luciferin is significant not only for binding affinity but also for selective molecular recognition of the S protein of SARS-CoV-2 in the BCL-based assay system.

The luminescence intensity of *Cypridina* luciferin is proportional to the trimeric-S-protein concentration in the range of 0–50 μg/mL and reaches a plateau above 50 μg/mL (Figure 5b). Given that *Cypridina* luciferin can emit light only with S protein but not with other salivary proteins, we next assessed the luminescence activity of the trimeric S protein spiked in 10% human saliva. We found that *Cypridina* luciferin reacted specifically with the S protein in human saliva as well as in the buffer system, with a linear response in the S-protein concentration range of 2.5–30 μg/mL (Figure 5c). The detection limits of the buffer system (0.92 μg/mL = 2.1 nM) and saliva system (0.94 μg/mL = 2.2 nM) were also in excellent agreement (Figure 5c). A comparison with a commercially available ELISA for the trimeric S protein showed that this BCL system can quantitatively detect the S protein in saliva with the same accuracy as an antibody-based assay (Figure 5d). Furthermore, the BCL system could detect the S protein spiked in more concentrated human saliva (50%) without any inhibition by saliva components (Figure S8).

Thus, the BCL-based assay system can detect the S protein of SARS-CoV-2 in a “mix-and-read” manner, in which luciferin is added to the untreated specimen, and the luminescence signal produced by the enzymatic reaction with the target protein is read for only 1 min. While LFA allows point-of-care (POC) COVID-19 diagnosis that meets the ASSURED criteria of the World Health Organization (WHO), the BCL system can detect the S protein more rapidly and with an accuracy comparable to that of the LFA method with S-protein-binding sialic acid^27^ (Table S7). Recently, hand-held-type luminometers have become commercially available, and it can be expected that the BCL system can be used as a new tool for POC diagnosis when combined with these instruments.

## Conclusions

This work revealed the pseudoluciferase activity of the SARS-CoV-2 S protein (wild type), which is a key protein in its viral entry into cells and a biomarker for diagnosis, using *Cypridina* luciferin; this pseudoluciferase activity, resulting in BCL signals, may potentially be used for the quantitative analysis of SARS-CoV-2 S protein in human saliva.

Out of the 36 luciferins tested, the monomeric S protein shows luminescence with only *Cypridina* luciferin. The luminescence reaction occurred with the full-length S protein but was less efficient with S fragment proteins such as the S1 and S2 subunits. The S proteins form a trimeric structure on the surface of SARS-CoV-2 virus particles. The trimeric S protein showed a higher catalytic efficiency by a factor of 2.7 than the monomeric one and required a larger amount of luciferin than the monomeric structure to reach the steady state in the enzymatic reaction. This is due to the interfaces between the units; the trimeric S protein has more interfaces than the monomeric S protein, which may act as the binding sites for *Cypridina* luciferin. Based on comparative experiments with *Cypridina* luciferin analogues, the 3-indolyl substituent at C-6 is involved in the luminescence activity, whereas the 3-(1-guanidino)propyl group plays an important role in the binding affinity. In addition, a docking simulation of *Cypridina* luciferin and its analogue with the trimeric S protein supported the notion of multiple luciferin binding and the superiority of the aforementioned functional groups in the enzymatic luminescence reaction of the SARS-CoV-2 S protein. Taking advantage of the reaction specificity of *Cypridina* luciferin toward the S protein, we successfully developed a new protein-analysis technique that can detect S proteins in human saliva in one minute without sample pretreatment.

## Supporting Information Available

Materials and methods; synthetic procedures for all luciferins; chemical structures of luciferins used in this work; structure of the SARS-CoV-2 S protein in trimeric form; luminescence profiles of luciferins with S proteins or *Cypridina* luciferase; binding affinities and binding poses of luciferin with the trimeric S protein predicted by the docking-simulation; BCL-based assay of trimeric S protein in saliva; NMR spectra of CLA1–3; references (PDF)
Transparent Peer Review report available (PDF)
