# Supplementary material for: Pseudo-Luciferase Activity of the SARS-CoV-2 Spike Protein for Cypridina Luciferin
Source: ACS Cent Sci. 2024 Jan 17;10(2):283–90. doi: 10.1021/acscentsci.3c00887 (PMC10906034; doi:10.1021/acscentsci.3c00887)
Supplement: Supplementary file 1 — oc3c00887_si_001.pdf [file oc3c00887_si_001.pdf]

Supporting Information for

Pseudo-Luciferase Activity of the SARS-CoV-2 Spike Protein for *Cypridina* Luciferin

Ryo Nishihara,[*,†,‡] Hisham M. Dokainish,[§,¶] Yoshiki Kihara,[†,#] Hiroki Ashiba,[†] Yuji Sugita,[¶,‖,⊥] and Ryoji Kurita[*,†,#]

[†]National Institute of Advanced Industrial Science and Technology (AIST), 1-1-1 Higashi, Tsukuba, Ibaraki 305-8566, Japan
[‡] Japan Science and Technology Agency (JST), PREST, 4-1-8, Honcho, Kawaguchi, Saitama 332-0012, Japan
[§] Faculty of Pharmaceutical Sciences, Hokkaido University, Nishi 6 Kita12 Kita-ku, Sapporo 060-0812, Japan
[¶]Theoretical Molecular Science Laboratory, RIKEN Cluster for Pioneering Research, 2-1 Hirosawa, Wako, Saitama 351-0198, Japan
[#]Faculty of Pure and Applied Sciences, University of Tsukuba, 1-1-1 Tennodai, Tsukuba, Ibaraki 305-8573, Japan
[‖] Laboratory for Biomolecular Function Simulation, RIKEN Center for Biosystems Dynamics Research, 6-7-1 Minatojima-minamimachi, Chuo-ku, Kobe, Hyogo 650-0047, Japan
[⊥]Computational Biophysics Research Team, RIKEN Center for Computational Science, 6-7-1 Minatojima-minamimachi, Chuo-ku, Kobe, Hyogo 650-0047, Japan

* To whom correspondence should be addressed: r.nishihara@aist.go.jp (R.N.); r.kurita@aist.go.jp (R.K.)

Contents

1. Experimental details S2
2. Supporting figures and tables S6
3. References S13

1. Experimental Procedures

1.1 Materials

1.1.1 Chemicals

SARS-CoV-2 (2019-nCoV) Spike S1+S2 ECD (R683A, R685A, F817P, A892P, A899P, A942P, K986P, V987P)-His Recombinant Protein, SARS-CoV-2 (2019-nCoV) Spike S1-His Recombinant Protein, SARS-CoV-2 (2019-nCoV) Spike S2 ECD(708S-1207E)-His Recombinant Protein, SARS-CoV-2 (2019-nCoV) Spike RBD-His Recombinant Protein, SARS-CoV-2 (2019-nCoV) Spike RBD(Y453F)-His Recombinant Protein, SARS-CoV-2 (2019-nCoV) Spike RBD(N501Y)-His Recombinant Protein, SARS-CoV Spike/S1 Protein (S1 Subunit, mFc Tag), MERS-CoV Spike/S1 Protein (S1 Subunit, aa 1-725, His Tag), and SARS-CoV-2 (BA.2.12.1) Spike S1+S2 trimer Protein (ECD, His Tag) were obtained from Sino Biological, Inc. (Beijing, China). Trimeric SARS-CoV-2 Spike Protein (full length) was obtained from Bio-Serv Co. at a concentration of 0.936 mg/mL in 20 mM HEPES buffer (pH = 7.5) containing 150 mM NaCl and 0.001% LMNG (Flemington, NJ, USA). Human normal saliva (HIV, HBV, HCV negative, informed consent obtained, from COVID-19 PCR-negative donor) was obtained from BiomedicaCRO Co. (Kyiv, Ukraine). IgA from human serum, mucin from pig stomach, α-amylase, lysozyme human, epidermal growth factor human, coelenterazine, and coelenterazine-h were obtained from Wako Pure Chemical Industries, Ltd. (Osaka, Japan). Lactoferrin human was obtained from Sigma-Aldrich Co. (St. Louis, MO, USA). SARS-CoV-2 Spike Trimer Specific ELISA kit was obtained from ACROBiosystems Co. Ltd. (Delaware, USA). *Cypridina* luciferin was obtained from Atto Co. (Tokyo, Japan). e-Coelenterazine, coelenterazine-f, coelenterazine-v, Prolume Purple, Prolume Purple II, Prolume Purple III, Prolume Purple IV, and Prolume Purple V were obtained from NanoLight Technologies, Ltd. (Pinetop, AZ, USA). Twenty-seven IPT luciferins (DeepBlueC, MCLA, BBlue1.1, BBlue1.2, BBlue1.3, BBlue1.4, BBlue1.5, BBlue1.6, BBlue1.7, BBlue1.8, BBlue2.1, BBlue2.2, BBlue2.3, BBlue2.4, BBlue3.1, BBlue3.2, BBlue3.3, BBlue3.4, BBlue3.5, BBlue3.6, 6-pi-Ph-CTZ, 6-piOH-2OH-CTZ, 6-piOH-2H-CTZ, HuLumino1) were synthesized according to literature procedures[1].

1.1.2 Preparation of stock solutions

S tock solutions of protein or luciferin were prepared as follows: monomeric S protein (0.1 mg/mL = 722 nM) in Milli-Q water; trimeric S protein (BA.2.12.1) (0.29 mg/mL = 722 nM) in Milli-Q

water; the salivary proteins (0.1 mg/mL) in Milli-Q water; luciferin solution (2 mM) in methanol. These solutions were diluted to appropriate concentrations using 1× phosphate buffered saline (PBS) buffer (pH = 7.4) or 10 mM 4-(2-hydroxyethyl)-1-piperazineethanesulfonic acid (HEPES) buffer (pH = 7.4). Protein concentrations were determined based on the absorbance at 280 nm using a spectrophotometer (NanoDrop OneC; Thermo Fisher Scientific, Inc.).

1.1.3 Synthesis and characterization of the Cypridina luciferin analogue (CLA) series

General: All reagents and solvents for organic synthesis were purchased from common commercial suppliers (Tokyo Kasei, Sigma Aldrich, or FUJIFILM Wako Pure Chemical) and were used without purification. All moisture-sensitive reactions were carried out under an argon atmosphere. The composition of mixed solvents is given as the volume ratio (v/v). ^{1}H NMR spectra were recorded on an Avance III-500 (Bruker Ltd.) spectrometer at room temperature. The ^{1}H NMR measurements were performed at 500 MHz. All chemical shifts are expressed relative to tetramethylsilane (δ = 0.0 ppm) as the internal standard or residual undeuterated solvent peaks (CHD_2OD in CD_3OD: δ = 3.31 ppm for ^{1}H); all coupling constants are given in Hz.

General synthetic procedure for the CLA series: Pyruvaldehyde (2 eq.) in ethanol (2.0 mL) and Milli-Q water (0.2 mL) was added to the amino-pyrazine analogue (0.13–0.19 mmol), before the mixture was stirred at room temperature (RT). After vacuum deaeration, the solution was cooled to 0 °C and hydrochloric acid (0.1 mL) was added. Once the solution reached RT, it was heated and stirred for 3 h at 80 °C. Then, the solvent was removed under reduced pressure, and the crude material was purified by column chromatography on silica gel (eluent composition: dichloromethane/methanol = 20/1), affording the luciferin as a yellow or brown solid. This synthetic scheme is a slight modification of a reported one[2].

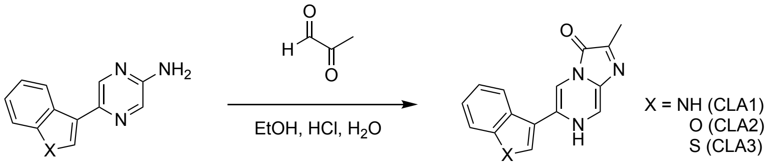

Scheme S1. Synthesis scheme of the CLAs.

CLA1 (yield: 43.1%): ^{1}H NMR (500 MHz, CD_3OD): δ (ppm) = 7.79 (d, J = 7.85 Hz, 1H), 7.74 (s, 1H), 7.62 (s, 1H), 7.58 (s, 1H), 7.37 (d, J = 8.05 Hz, 1H), 7.08–7.14 (m, 2H), 2.35 (s, 3H).

CLA2 (yield: 57.8%): ^{1}H NMR (500 MHz, CD_3OD): δ (ppm) = 8.14 (s, 1H), 7.88-7.90 (m, 2H),

7.82 (s, 1H), 7.51 (d, J = 7.90 Hz, 1H), 7.30–7.34 (m, 2H), 2.35 (s, 3H).

CLA3 (yield: 30.5%): ^{1}H NMR (500 MHz, CD3OD): δ (ppm) = 7.86–7.95 (m, 4H), 7.63 (s, 1H), 7.35–7.41 (m, 2H), 2.37 (s, 3H).

1.2 Luminescence assay

1.2.1 Luminescence intensities of 36 IPT luciferins with the monomeric S protein (full length) of SARS-CoV-2

To 5 μL of an aliquot of the monomeric S protein (722 nM) in a 384-well microplate (PerkinElmer, Massachusetts, USA), 45 μL of 20 μM luciferin in PBS buffer (pH = 7.4) was added, and the luminescence signals were read immediately for 60 s using a plate reader (Glomax® Explorer; Promega, Wisconsin, USA).

1.2.2 Luminescence intensities of luciferins with the trimeric S protein (full length) of SARS-CoV-2

To 5 μL of an aliquot of the trimeric S protein (722 nM) in a 96-well microplate (PerkinElmer), 45 μL of 20 μM luciferin in PBS buffer (pH = 7.4) was added using an injector, and the luminescence signals were read immediately for 60 s using a plate reader (Glomax® Explorer).

1.2.3 Kinetic parameters (K_m and V_{max}) of luciferin with S protein

To 5 μL of an aliquot of the monomeric or trimeric S protein (722 nM) in a 96-well microplate (PerkinElmer), 45 μL of 0–100 μM or 0-50 μM luciferin in PBS buffer (pH = 7.4) was added using an eight-channel pipette, and the luminescence signals were read immediately for 60 s using a plate reader (Spark Cyto; TECAN, Männedorf, Switzerland) with the following settings: mode, kinetic; kinetic cycles, 60 or 30; integration time, 1 s. The kinetic parameters (K_{m} and V_{max}) were calculated by fitting the total luminescence intensity for 30 s, excluding the background signals obtained with only luciferin, using the Michaelis–Menten equation in GraphPad Prism 9.

1.2.4 Luminescence spectra

To 5 μL of an aliquot of the protein or organic solvent in a 96-well microplate (PerkinElmer), 45 μL of 20 μM luciferin in PBS buffer (pH = 7.4) was added using an injector, and the luminescence spectra were read immediately using a plate reader (Spark Cyto) with the following settings: integration time, 1 s; central wavelength start, 398 nm; and central wavelength end, 653 nm.

1.2.5 Docking simulation

All docking simulations were performed using AutoDock Vina Software, V1.2.3[3]. The cluster centers of S protein's Down-to-Up transitions from the previous MD simulations[4] were used as receptors. Multiple conformations of Spike including, Down, Down-like and Down-with-slightly-increased-hinge-angle (I1) of the receptor binding domain (RBD) were used. All seven conformations of the trimeric S protein are glycosylated. The two ligands (Luciferin and CLA1) were constructed, and energy-minimized using the Merck molecular force field (MMFF94s) as implemented in ChemDoodle 3D software[5]. Two docking steps were performed. Initially, the whole protein was considered as a receptor (blind docking), using a large box size of 140 x 126 x 126 Å^3 with a grid spacing of 1.00 Å. The monomeric S protein was located in a box the size of 140 x 94 x126 Å^3. The top-20 poses were generated while based on docking scores, and only the top ten were used for further analysis. Based on the blind-docking results, refined docking with a default grid spacing of 0.375 Å were performed, using six potential receptor regions. This includes the N-terminal domain (NTD), NTD/RBD interface, small subdomain 1 (SD1)/ subunit 2 (S2) interface, and the three regions of S2/S2/S2 including top and bottom interfaces as well as the S2 outer region. The top-three poses from all six docking simulations (72 poses) of the S protein Down conformations (symmetric and asymmetric) were further analyzed and energy averaged. The PyMOL software was used to investigate all binding poses interactions and visualization[6].

1.2.6 Luminescence intensities of luciferin with the salivary proteins

To 5 µL of an aliquot of the salivary proteins (0.1 mg/mL) in a 96-well microplate (PerkinElmer), 45 µL of 20 µM *Cypridina* luciferin in PBS buffer (pH = 7.4) was added using an injector, and the luminescence signals were read immediately for 60 s using a plate reader (Glomax® Explorer).

1.2.7 SARS-CoV-2 trimeric S protein assay in human saliva

A human saliva sample was centrifuged at 1000*g* for 10 min at 4 °C to remove floating material according to a reported procedure[7]. In the luminescence assay, to 5 µL of an aliquot of 10% human saliva spiked with the trimeric S protein (25–250 µg/mL) in a 96-well microplate (PerkinElmer), 45 µL of 20 µM *Cypridina* luciferin in HEPES buffer (pH = 7.4) was added using an injector, and the luminescence signals were read immediately for 60 s using a plate reader (Glomax® Explorer). To calculate the concentration of S protein in the saliva from the luminescence signals, we used a calibration curve obtained by measurement in a buffer system spiked with the same

concentration of protein. In the ELISA, trimeric S protein from Bio-Serv, Co. was used as standard sample and the concentration of S protein in saliva was measured according to the manufacturer's protocol.

2. Supporting figures and tables

2.1 Chemical structures of luciferins

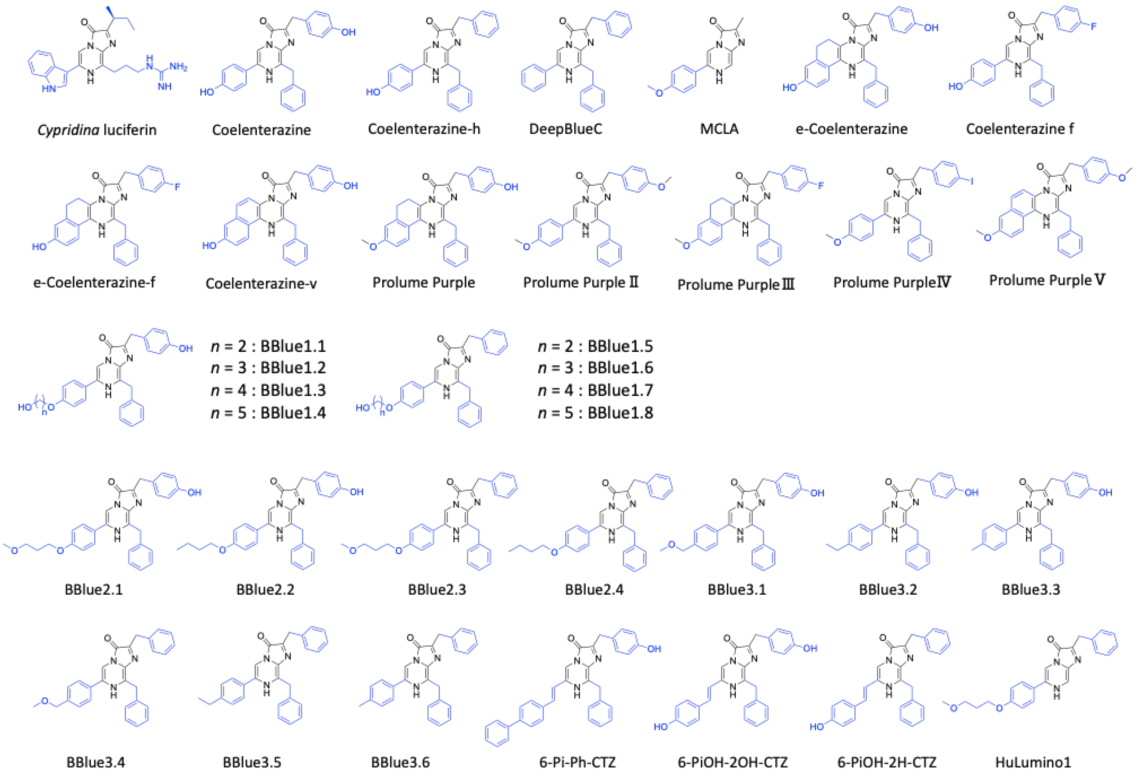

Figure S1. Chemical structures of IPT luciferins with different C-2, C-5, C-6, and C-8 substituents used in this study.

2.2 Luminescence profiles

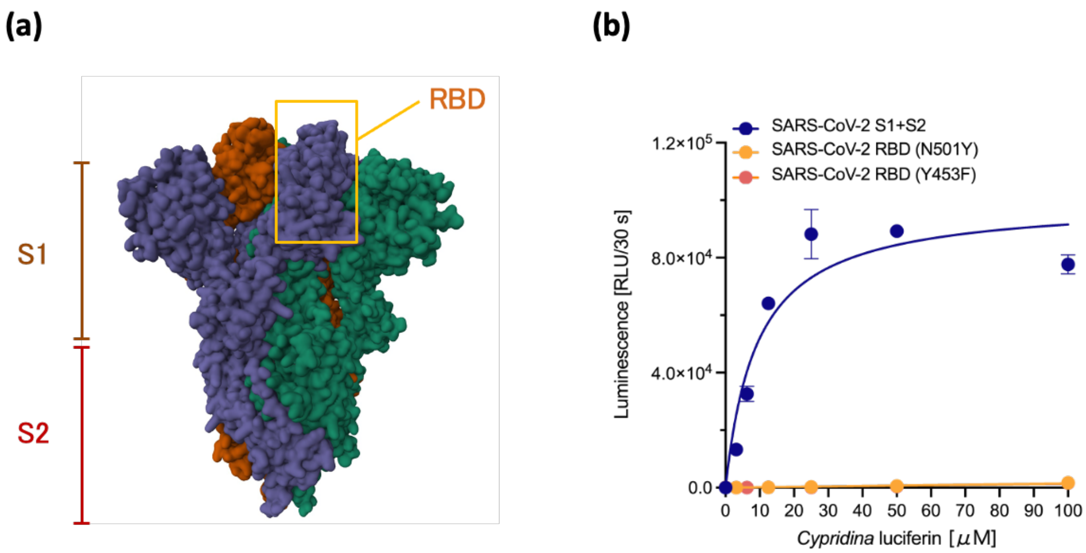

Figure S2. (a) Structure of the SARS-CoV-2 S protein (trimer; PDB code 6VXX). The S protein has two subunits in each monomer, i.e., S1, which includes the RBD, and S2. (b) Dose-dependent luminescence intensities: *Cypridina* luciferin (0-100 μM) in the presence of the monomeric S protein (72 nM).

Table S1. V_{max} values of *Cypridina* luciferin with the S1+S2 full-length protein or fragment proteins

	K_m [μM]	V_{max} [$\times 10^5$ RLU/30 s]
SARS-CoV-2 S1+S2	9.28 ± 1.03	1.00 ± 0.02
SARS-CoV-2 S1	91.1 ± 21.1	0.38 ± 0.06
SARS-CoV-2 S2	42.2 ± 8.76	0.32 ± 0.05
SARS-CoV-2 RBD	N.D.	N.D.
SARS-CoV-2 RBD (N501Y)	N.D.	N.D.
SARS-CoV-2 RBD (Y453F)	N.D.	N.D.

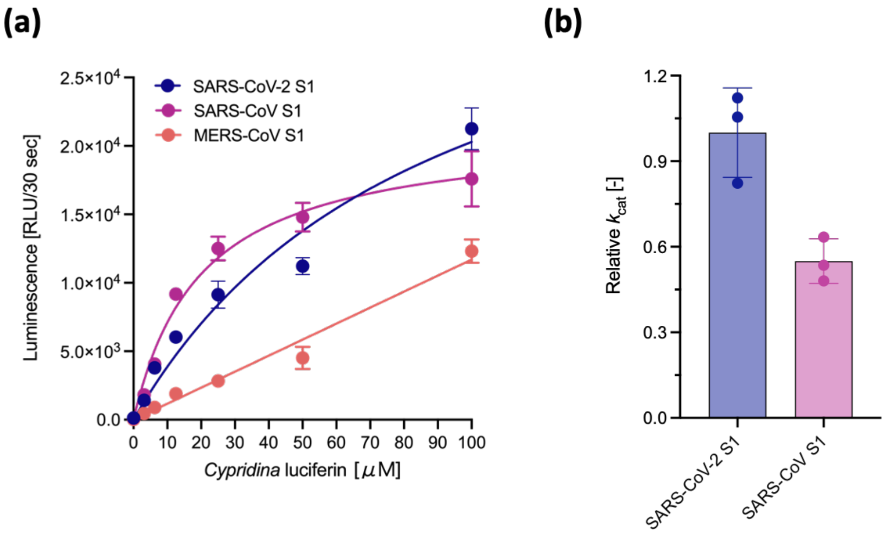

Figure S3. (a) Dose-dependent luminescence intensities: *Cypridina* luciferin (0-100 μM) in the presence of coronavirus S1 protein (72 nM). (b) The relative k_{cat} value was calculated by normalizing the V_{max} value of the *Cypridina* luciferin/SARS-CoV-2 S1 protein (72 nM) pair to 1.0. Error bars represent the standard deviation of three independent measurements.

Table S2. V_{max} values of *Cypridina* luciferin with the fragment proteins

	K_m [μM]	V_{max} [×10^5 RLU/30 s]
SARS-CoV-1 S1	20.2 ± 4.79	0.21 ± 0.03
MERS-CoV-1 S1	N.D.	N.D.

Table S3. V_{max} values of *Cypridina* luciferin with the trimeric S protein

	K_m [μM]	V_{max} [×10^5 RLU/30 s]
Trimeric S	15.0 ± 3.61	2.74 ± 0.31

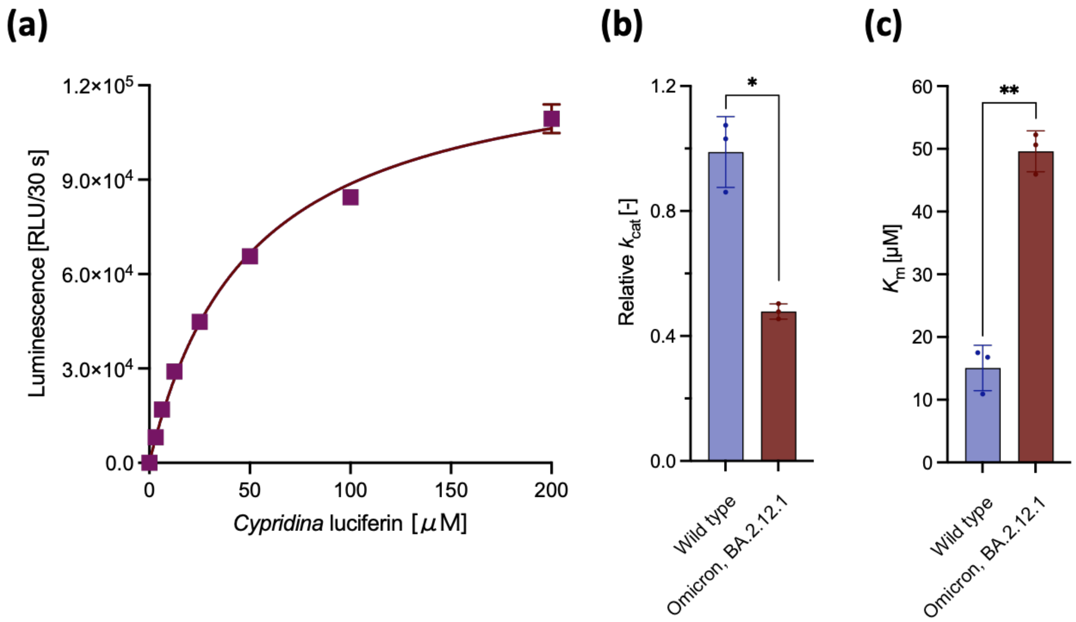

Figure S4. (a) Dose-dependent luminescence intensity of *Cypridina* luciferin (0-200 μM) in the presence of the trimeric protein of SARS-CoV-2 variant (omicron, BA2.12.1) (72 nM). (b) The relative k_{cat} value was calculated by normalizing the V_{max} value of the *Cypridina* luciferin/trimeric S protein (wild type) (72 nM) pair to 1.0. $^{*}P < 0.02$ (t-test). (c) K_m values of the trimeric S proteins for *Cypridina* luciferin. $^{**}P < 0.0001$ (t-test). Error bars represent the standard deviation of three independent measurements.

Table S4. V_{max} values of *Cypridina* luciferin with the trimeric S protein (BA.2.12.1)

	K_m [μM]	V_{max} [×10^5 RLU/30 s]
Trimeric S (BA.2.12.1)	49.6 ± 3.26	1.32 ± 0.06

Table S5. V_{max} values of CLA1 with the monomeric S protein or the trimeric S protein

	K_m [μM]	V_{max} [×10^5 RLU/30 s]
SARS-CoV-2 S1+S2	8.23 ± 0.46	0.40 ± 0.00
Trimeric S	19.3 ± 2.46	2.77 ± 0.21

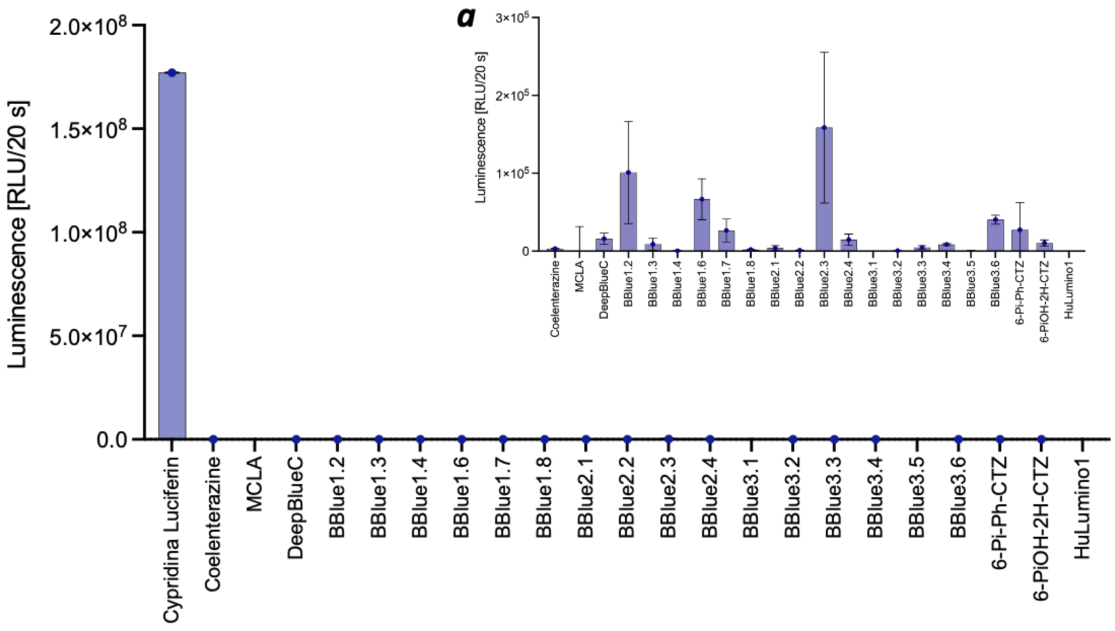

Figure S5. Luminescence response of *Cypridina* luciferase (38 ng/mL) treated with various IPT luciferins (2 μM) in 10 mM phosphate buffer (pH = 7.4). Inset *a* shows the luminescence intensities of luciferins without *Cypridina* luciferin. Error bars represent the standard deviation of three independent measurements.

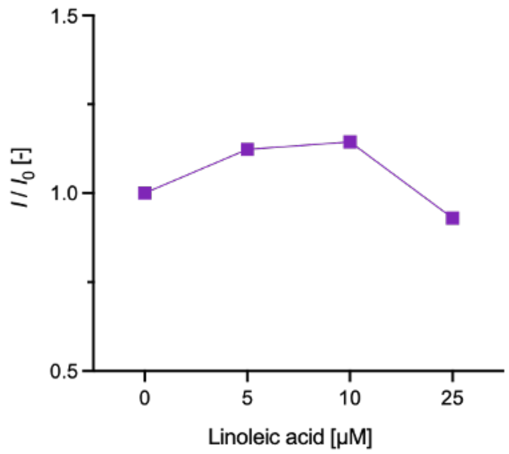

Figure S6. Effect of linoleic acid on the luminescence of *Cypridina* luciferin (20 μM) and the trimeric S protein (222 nM): Luminescence response in the presence (I) vs. the absence (I_0) of linoleic acid at different concentrations (0–25 μM).

2.3 Docking-simulation studies

Table S6. Comparison of the binding affinities of the top-ten-ranked poses of luciferin with the trimeric S protein

Luciferin	$D1_{Sym}$ [kcal/mol]	$D2_{Sym}$ [kcal/mol]	$D1_{Asym}$ [kcal/mol]	$D2_{Asym}$ [kcal/mol]	I1a [kcal/mol]	I1b [kcal/mol]	I1c [kcal/mol]	Average [kcal/mol]
Cypridina luciferin	-9.379	-8.827	-9.789	-9.789	-9.492	-8.445	-9.221	-9.278
CLA1	-8.397	-8.467	-8.649	-8.71	-8.707	-9.006	-8.946	-8.697

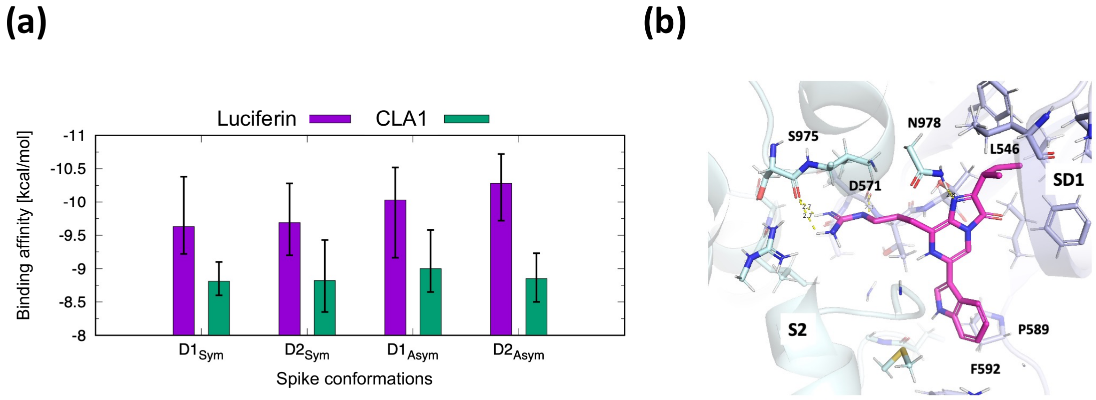

Figure S7. Predicted binding affinity and pocket. (a) Comparison of the average binding affinity using the top-three-ranked poses from six specified regions, including four interfaces (NTD/RBD, SD1/S2, S2/S2/S2 at the top region closer to RBD, S2/S2/S2 at the bottom region), the NTD domain, and the S2 unit. (B) Binding pose of *Cypridina* luciferin at the SD1/S2 interface in the symmetric D1 conformation.

2.4 BCL-based assay

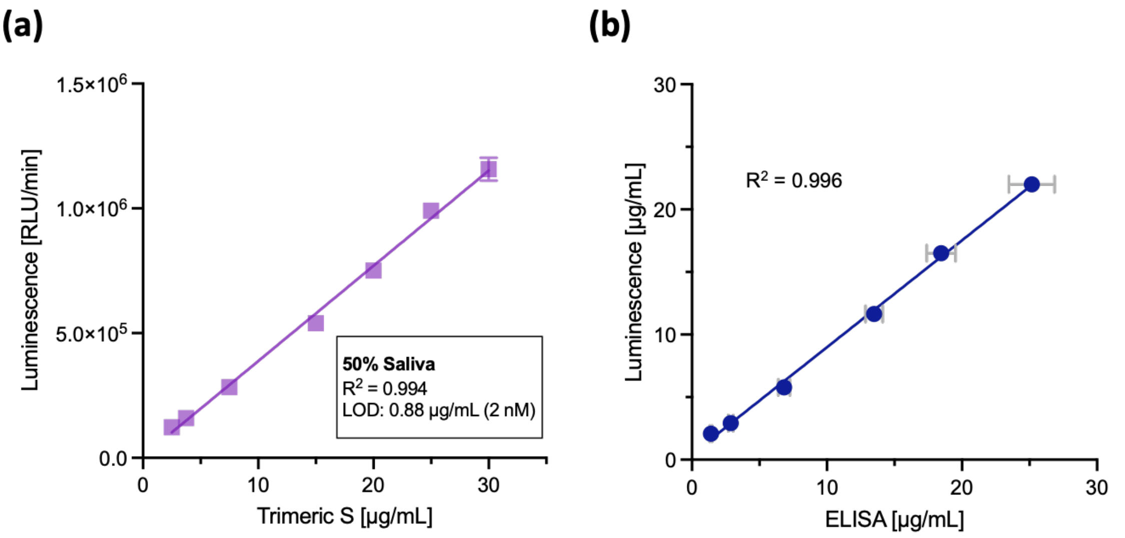

Figure S8. (a) Luminescence intensity of *Cypridina* luciferin (20 μM) in a saliva system (50%) that contains 0.5-30 μg/mL of the trimeric S protein. (b) Correlation between the measured concentrations of SARS-CoV-2 S protein using luminescence and ELISA. The markers and error bars represent the average and standard deviations of three independent measurements.

Table S7. Comparison with representative antigen tests

Targeted S protein	Material for S protein binding	Method for detection	Assay time	LOD
S1[8]	Sialic acid	LFA*	30 min	5 nM
RBD[9]	Antibody	LFA*	16 min	0.1 ng/mL (≈3.7 pM)
Trimeric S[10]	Aptamer	ALISA**	>3 h	2 nM
Trimeric S (***this work***)	Luciferin	BCL	1 min	2.1 nM

*LFA: lateral flow assay

**ALISA: aptamer-linked immobilized sorbent assay

2.5 NMR spectra

^{1}H NMR spectrum of CLA1 in CD_3OD

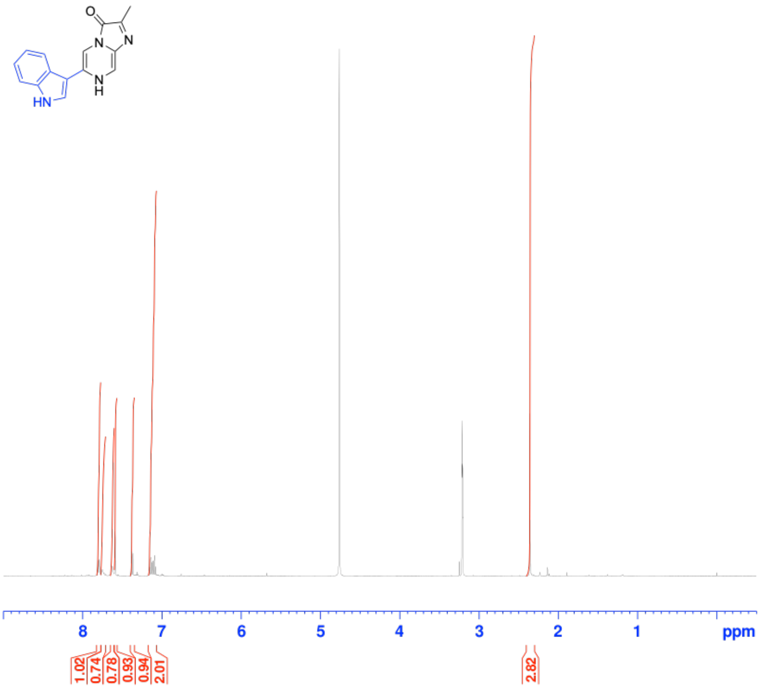

^{1}H NMR spectrum of CLA2 in CD_3OD

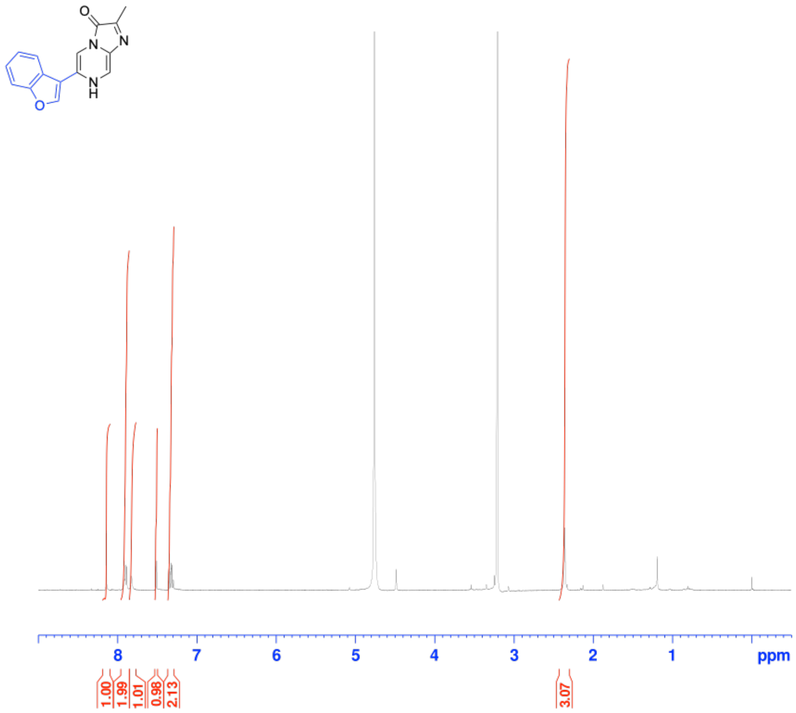

^{1}H NMR spectrum of CLA3 in CD_3OD

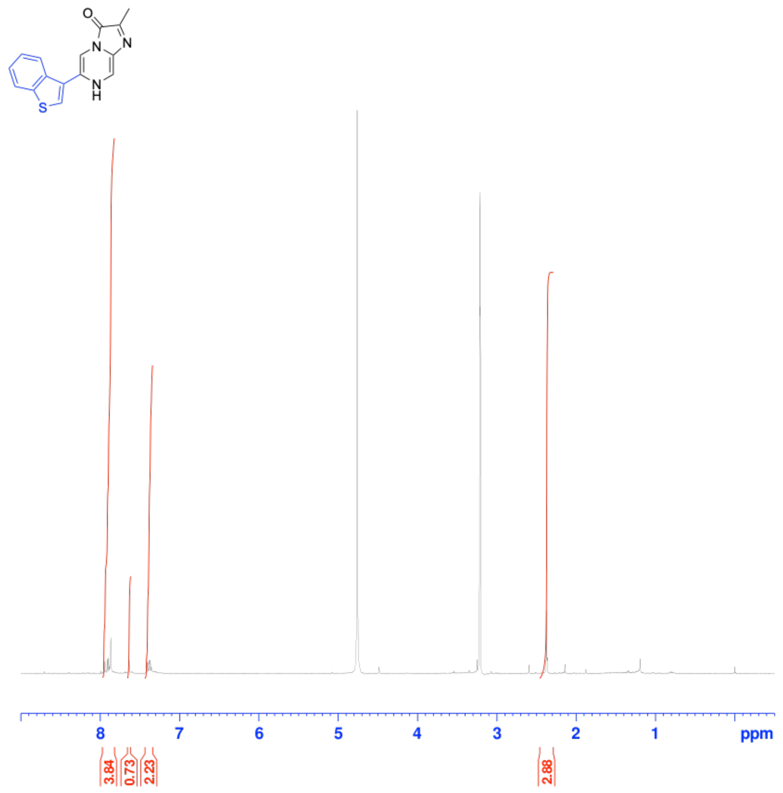

4. References

(1) Nishihara, R.; Paulmurugan, R.; Nakajima, T.; Yamamoto, E.; Natarajan, A.; Afjei, R.; Hiruta, Y.; Iwasawa, N.; Nishiyama, S.; Citterio, D.; et al. Highly bright and stable NIR-BRET with blue-shifted coelenterazine derivatives for deep-tissue imaging of molecular events in vivo. *Theranostics* **2019**, *9*, 2646-2661. Nishihara, R.; Abe, M.; Nishiyama, S.; Citterio, D.; Suzuki, K.; Kim, S. Luciferase-Specific Coelenterazine Analogues for Optical Contamination-Free Bioassays. *Sci. Rep.* **2017**, *7*, 908.

(2) Hirano, T.; Takahashi, Y.; Kondo, H.; Maki, S.; Kojima, S.; Ikeda, H.; Niwa, H. The reaction mechanism for the high quantum yield of Cypridina (Vargula) bioluminescence supported by the chemiluminescence of 6-aryl-2-methylimidazo[1,2-a]pyrazin-3(7H)-ones (Cypridina luciferin analogues). *Photochem. Photobiol. Sci.* **2008**, *7*, 197-207.

(3) Eberhardt, J.; Santos-Martins, D.; Tillack, A.; Forli, S. AutoDock Vina 1.2.0: New Docking Methods, Expanded Force Field, and Python Bindings. *J. Chem. Inf. Model.* **2021**, *61*, 3891-3898. Trott, O.; Olson, A. Software News and Update AutoDock Vina: Improving the Speed and Accuracy of Docking with a New Scoring Function, Efficient Optimization, and Multithreading. *J. Comput. Chem.* **2010**, *31*, 455-461.

(4) Dokainish, H.; Re, S.; Mori, T.; Kobayashi, C.; Jung, J.; Sugita, Y. The inherent flexibility of receptor binding domains in SARS-CoV-2 spike protein. *elife* **2022**, *11*, e75720.

(5) ChemDoodle 3D: 3D Chemical Graphics, Animations and Modeling (Version6.6.0), iChemLabs,. Available from https://www.chemdoodle3d.com.

(6) Schrodinger LLC (2021) The PyMOL Molecular Graphics System, Version 2.4 PyMOL.

(7) Isho, B.; Abe, K.; Zuo, M.; Jamal, A.; Rathod, B.; Wang, J.; Li, Z.; Chao, G.; Rojas, O.; Bang, Y. Persistence of serum and saliva antibody responses to SARS-CoV-2 spike antigens in COVID-19 patients. *Sci. Immunol.* **2020**, *5*, eabe5511.

(8) Baker, A.; Richards, S.; Congdon, T.; Hasan, M.; Guy, C.; Zwetsloot, A.; Gallo, A.; Lewandowski, J.; Stansfeld, P.; Straube, A. The SARS-COV-2 Spike Protein Binds Sialic Acids, and Enables Rapid Detection in a Lateral Flow Point of Care Diagnostic Device. *ACS Cent. Sci.* **2021**, *7*, 379-380.

(9) Liu, D.; Ju, C.; Han, C.; Shi, R.; Chen, X.; Duan, D.; Yan, J.; Yan, X. Nanozyme chemiluminescence paper test for rapid and sensitive detection of SARS-CoV-2 antigen. *Biosens. Bioelectron.* **2021**, *173*, 112817.

(10) Gupta, A.; Anand, A.; Jain, N.; Goswami, S.; Anantharaj, A.; Patil, S.; Singh, R.; Kumar, A.; Shrivastava, T.; Bhatnagar, S. A novel G-quadruplex aptamer-based spike trimeric antigen test for the detection of SARS-CoV-2. *Mol. Ther. Nucleic. Acids* **2021**, *26*, 321-332.
