# Supplementary material for: Pseudo-Luciferase Activity of the SARS-CoV-2 Spike Protein for Cypridina Luciferin
Source: ACS Cent Sci. 2024 Jan 17;10(2):283–90. doi: 10.1021/acscentsci.3c00887 (PMC10906034; doi:10.1021/acscentsci.3c00887)
Supplement: Supplementary file 2 — oc3c00887_si_002.pdf [file oc3c00887_si_002.pdf]

oc-2023-00887p.R1

Name: Peer Review Information for "Pseudo-Luciferase Activity of the SARS-CoV-2 Spike Protein for <i>Cypridina</i> Luciferin"

First Round of Reviewer Comments

Reviewer: 1

Comments to the Author

In the current manuscript, Ryo Nishihara et al. report the unique pseudo-luciferase activity of SARS-CoV-2 Spike protein. On the basis of this finding, it is expected to detect the SARS-CoV-2 S protein via bioluminescence assay easily. I agree to accept this manuscript for publication in this journal after adding more discussion or results from the two aspects below:

1. Since Cypridina luciferin binds to S protein, can it inhibit the entry of SARS CoV-2 into cells?

2. There are several types of small molecules that are reported to bind S protein. Is their binding site(s) the same as that of Cypridina luciferin and S protein. Is it possible to use the bioluminescence assay for S protein-binding compound screening?

Reviewer: 2

Comments to the Author

The article by Nishihara and colleagues describe the identification of a luciferin variant that is specifically cleaved by the spike protein of SARS-CoV-2. The authors have carefully tested a large number of luciferin variants and supported their findings with detailed kinetic studies of the catalytic activity of individual spike protein subunits and the trimer, as well as docking simulations. The luciferin from Cypridina showed the highest signal-to-noise ratio of 35. This study may be potentially very important as a simple detection scheme for Covid-19 infections, thus it is recommended for publication.

Although the authors investigated the bioluminescent detection of the S protein spiked in saliva it seems that rather high amounts of the S protein (µg/mL range) are needed for a specific detection. The authors should determine the limit of detection for the determination of the S protein. Typically, good tests should aim for a limit of detection, which is at least 1000 times lower than 1 µg/mL. The S protein was

spiked in 10% saliva. Why was it not possible to do the tests in a more concentrated saliva sample (e.g. 50%)? The less diluted the sample is the lower are the concentrations in the original sample that can be determined. Are there more interferences when using higher amounts of saliva? It is also known that the S protein is more variable as compared e.g. the nucleoprotein. Do all S proteins of the different variants of the SARS-CoV-2 (WT, beta, lamba, omicron…) show the same catalytic activity with Cypridina luciferin? It should also be noted from which virus variant the S protein is that the authors have selected. The performance needs to be critically discussed in the manuscript – this will not diminish the high quality of the results.

For the diagnosis of an infection, it would be necessary detect the whole virus, not the isolated S proteins spiked in saliva. The authors should test real samples of infected patients or – if real samples are not available – they could by the inactivated virus and spice this into saliva. Then they will also see if the whole virus shows catalytic activity or if a lysis buffer is needed to disassemble the virus and release the S protein.

Author's Response to Peer Review Comments:

National Institute of Advanced Industrial Science and Technology (AIST)
1-1-1 Higashi, Tsukuba, Ibaraki 305-8566, Japan
Tel: +81-29-861-6158 / Fax: +81-29-861-6177
E-mail: r.nishihara@aist.go.jp / r.kurita@aist.go.jp

November, 22, 2023

Submission of revised manuscript ID oc-2023-00887p

Prof. Dr. Editor
Senior Editor
ACS Central Science

Dear Prof. Editor,

Thank you for considering our manuscript for publication in *ACS Central Science* and for forwarding the referees' comments to us. We also gratefully acknowledge the efforts of the referees in providing us with critical and helpful comments.

We would hereby like to submit a revised version of our manuscript for further processing. Taking into consideration the reviewers' comments, we have revised our originally submitted manuscript and the supporting information. We have also added additional experimental data where necessary. In reply to the editor's comments, we have formatted the manuscript and the supporting information, and we have also added a note regarding potentially competing financial interests.

On the following pages, you will find our point-by-point responses to the referees' requests. Comments referring to changes made in the manuscript or the supporting information are marked in red. In addition to two new 'clean' files in Word (main text) and PDF (supporting information) format, we provide annotated PDF files with all amendments visualized for your convenience.

We would like to kindly ask you to consider our revised manuscript for publication. We hope that you will find our amendments satisfactory. In case you have any further questions, please feel free to contact us at any time.

If this manuscript should be accepted for publication, we would like to announce it in a press release, and therefore, please do not publish online or otherwise immediately upon acceptance.

Yours sincerely,

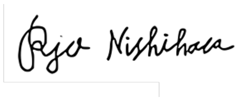

Ryo Nishihara and Ryoji Kurita
National Institute of Advanced Industrial Science and Technology
E-mail: r.nishihara@aist.go.jp/r.kurita@aist.go.jp

National Institute of Advanced Industrial Science and Technology (AIST)
1-1-1 Higashi, Tsukuba, Ibaraki 305-8566, Japan
Tel: +81-29-861-6158 / Fax: +81-29-861-6177
E-mail: r.nishihara@aist.go.jp / r.kurita@aist.go.jp

Reply to the comments of Reviewer #1

Comment-1: Since Cypridina luciferin binds to S protein, can it inhibit the entry of SARS CoV-2 into cells?

Reply-1:

For the following reason, we envision that *Cypridina* luciferin may not inhibit the entry of SARS-CoV-2 into cells. The first step of viral entry into cells is binding of the receptor binding domain (RBD) in the S protein to the host-cell surface receptor, i.e., angiotensin-converting enzyme-2 (ACE2). Based on this pathogenesis of SARS-CoV-2 infections, the inhibition of the binding of the RBD to ACE2 is one of the approaches to prevent the entry of the virus. Using a cryo-EM, Toelzer *et al.* have already revealed that linoleic acid (LA) tightly binds the cavities in the interfaces between RBDs, which reduces the RBD/ACE2 interaction (*cf. Science*, **2020**, *370*, 725-730). However, blocking the RBD/RBD interface pocket with LA does not inhibit the luminescence reaction of *Cypridina* luciferin (*cf.* Figure S5 in our manuscript) Furthermore, the luciferin does not react with the RBD itself and its variants (*cf.* Figure 1d and S2 in our manuscript). These results indicate that the RBD/RBD interface and the RBD itself are not binding sites for the luciferin. Therefore, at least *Cypridina* luciferin is unlikely to inhibit the RBD/ACE2 interaction in the first step of viral entry.

Comment-2: There are several types of small molecules that are reported to bind S protein. Is their binding site(s) the same as that of Cypridina luciferin and S protein. Is it possible to use the bioluminescence assay for S protein-binding compound screening?

Reply-2:

Most studies regarding S-protein-binding molecules have targeted the binding between the small molecules and the RBD (*cf.* e.g., *ACS Cent. Sci.*, **2023**, *9*, 252-265; *Science*, **2020**, *370*, 725-730; *Comput. Biol. Med.*, **2021**, *136*, 104631). Our docking studies, which use different conformations of the S protein, predicted the possible binding sites of *Cypridina* luciferin, particularly at the domain interfaces (RBD/NTD, SD1/S2 and S2/S2), which might have no effect regulating RBD dynamics or RBD/ACE2 binding. Therefore, *Cypridina* luciferin cannot be used for RBD-binding compound screening.

Moreover, it is not clear whether all predicted binding sites are involved in the oxidative (luminescence) reaction of luciferin. However, if the binding site(s) where the luminescence reaction occurs could be determined experimentally, it would be possible to screen compounds that bind to that site.

National Institute of Advanced Industrial Science and Technology (AIST)
1-1-1 Higashi, Tsukuba, Ibaraki 305-8566, Japan
Tel: +81-29-861-6158 / Fax: +81-29-861-6177
E-mail: r.nishihara@aist.go.jp / r.kurita@aist.go.jp

Reply to the comments of Reviewer #2

Comment-1: Although the authors investigated the bioluminescent detection of the S protein spiked in saliva it seems that rather high amounts of the S protein (µg/mL range) are needed for a specific detection. The authors should determine the limit of detection for the determination of the S protein. Typically, good tests should aim for a limit of detection, which is at least 1000 times lower than 1 µg/mL.

Reply-1:

We have added the values of the limit of detection (LOD) for the determination of the trimeric S protein and have added a corresponding description in the revised main text as shown below.

"*The detection limit of the buffer system (0.92 µg/mL = 2.1 nM) and saliva system (0.94 µg/mL = 2.2 nM) were also in excellent agreement (Figure 5c).*" (Page 6, right column)

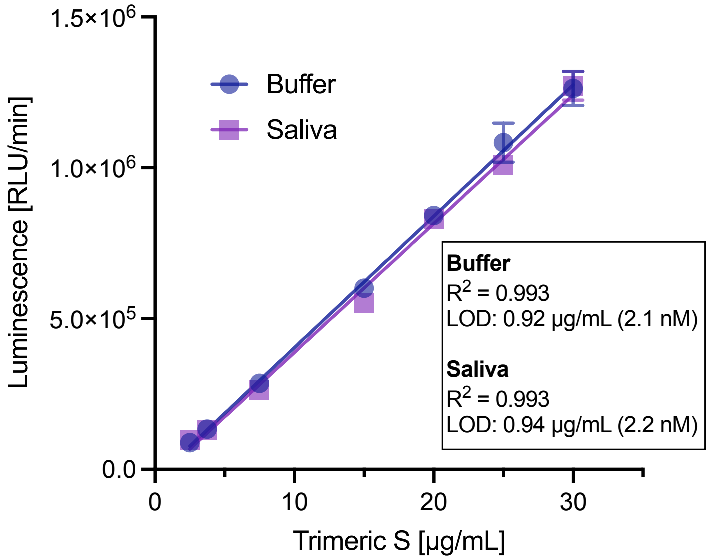

Figure 5. (c) Luminescence intensity of *Cypridina* luciferin (20 µM) in a buffer system or a saliva system that contain 0.5-30 µg/mL of the trimeric S protein.

In addition, the LOD value of our developed system (BCL) is compared to that of representative antigen tests. As summarized in Table S7 (added to the revised supporting information), the LOD value of the BCL-based assay is comparable to that of assays with S-protein-binding materials such as a lateral flow assay (LFA) with sialic acid (*cf. ACS Cent. Sci.*, **2020**, *6*, 2046-2052) and the aptamer-linked immobilized sorbent assay (ALISA) with the aptamer (*cf. Mol. Ther. Nucleic. Acids*, **2021**, *26*, 321-332). The LFA with S-protein-binding antibody exhibits an LOD value that is approximately by the orders of magnitude lower than that of assays with alternative materials (e.g., a luciferin) toward the antibody. However, the preparation of perfectly matched paired antibody to S protein is time-consuming and costly due to the development of the immune system with animals and the manufacturing process using cultured cells (*cf. TrAC*, **2021**, *145*, 116452). Furthermore, the assay time of LFA with antibody is relatively high (~16 min). In contrast, luciferin can be chemically synthesized, and react (detect) the targeted S protein much faster (~1 min).

Therefore, the luciferin-based S-protein-detection method may potentially find use as a good SARS-CoV-2 test that is cheaper and faster than antibody-based assays, with an LOD value comparable to those of previously reported representative assays using aptamers and sialic acid.

National Institute of Advanced Industrial Science and Technology (AIST)
1-1-1 Higashi, Tsukuba, Ibaraki 305-8566, Japan
Tel: +81-29-861-6158 / Fax: +81-29-861-6177
E-mail: r.nishihara@aist.go.jp / r.kurita@aist.go.jp

Table S7. Comparison with the representative antigen tests

Targeted S protein	Material for S protein binding	Method for detection	Assay time	LOD
S1	Sialic acid	LFA*	30 min	5 nM
RBD	Antibody	LFA*	16 min	0.1 ng/mL (≈3.7 pM)
Trimeric S	Aptamer	ALISA**	>3 h	2 nM
Trimeric S (***this work***)	Luciferin	BCL	1 min	2.1 nM

*LFA: lateral flow assay
**ALISA: aptamer-linked immobilized sorbent assay

Comment-2: The S protein was spiked in 10% saliva. Why was it not possible to do the tests in a more concentrated saliva sample (e.g. 50%)? The less diluted the sample is the lower are the concentrations in the original sample that can be determined. Are there more interferences when using higher amounts of saliva?

Reply-2:
Based on the reviewer's comment, we have performed additional experiments in 50% saliva containing the trimeric S protein (*cf.* Figure S8a) and compared the results with a commercially available ELISA (*cf.* Figure S8b). Thus, we were able to confirm that the BCL-based assay allows quantifying the spiked S proteins even in 50% saliva with LOD values comparable to that of the assay in 10% saliva. The obtained results indicate that high-concentrated saliva samples do not inhibit the luminescence reaction between the S protein and luciferin.
We have added Figure S8 to the revised supporting information, and a corresponding description to the revised main text as shown below:

"*Furthermore, the BCL system is able to detect the S protein spiked in more concentrated human saliva (50%) without any inhibition by saliva components (Figure S8).*" (Page 6, right column)

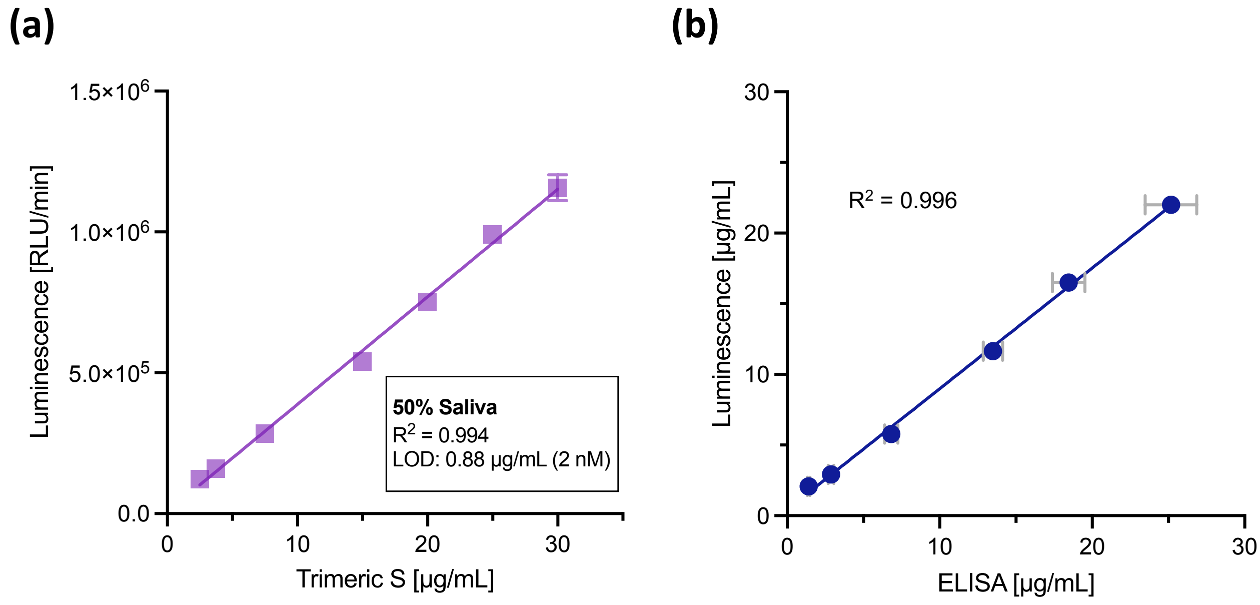

Figure S8 (a) Luminescence intensity of *Cypridina* luciferin (20 μM) in 50% saliva containing 0.5-30 μg/mL of the trimeric S protein. (b) Correlation between the measured concentrations of SARS-CoV-2 S protein using luminescence and ELISA. The markers and error bars represent the average and standard deviations of three independent measurements.

National Institute of Advanced Industrial Science and Technology (AIST)
1-1-1 Higashi, Tsukuba, Ibaraki 305-8566, Japan
Tel: +81-29-861-6158 / Fax: +81-29-861-6177
E-mail: r.nishihara@aist.go.jp / r.kurita@aist.go.jp

Comment-3: It is also known that the S protein is more variable as compared e.g. the nucleoprotein. Do all S proteins of the different variants of the SARS-CoV-2 (WT, beta, lamba, omicron…) show the same catalytic activity with Cypridina luciferin? It should also be noted from which virus variant the S protein is that the authors have selected. The performance needs to be critically discussed in the manuscript – this will not diminish the high quality of the results.

Reply-3:

We have addressed the luminescence characteristics of *Cypridina* luciferin with the trimeric S protein of SARS-CoV-2 (BA.2.12.1), one of the omicron variants, which is currently the dominant SARS-CoV-2 linage. Especially omicron (BA.2.12.1) has gained global attention due to its circulation among *fully vaccinated and boosted* individuals, which has been attributed to its characteristics with respect to antibody evasion and the high binding affinity toward the ACE2 receptor (*cf. Ann. Med. Surg.*, **2022**, *79*, 104034; *Science*, **2022**, *375*, 760-764.).

We have added the luminescence properties of the trimeric S protein of omicron (BA.2.12.1), together with a description in the revised main text as shown below.

"*The BCL reaction could also be observed in the trimeric S protein of a SARS-CoV-2 variant (Omicron, BA.2.12.1), catalyzing the luminescence reaction of Cypridina luciferin (Figure S4). This is due to the overall organization of the trimeric S protein, which is conserved between the wild-type and the omicron variant.*[20] *As a result of the amino-acid mutations, the catalytic efficiency of the omicron variant was reduced to half of that of the wild type, with a relatively high* K_m *value of 49.6 μM (Figure S4 and Table S4). Hence, amino-acid mutations have a significant effect on kinetic profiles, but other mutant S proteins, in which the structure of the trimeric S protein (wild type) is conserved, may also exhibit pseudo luciferase activity*." (Page 4, left column)

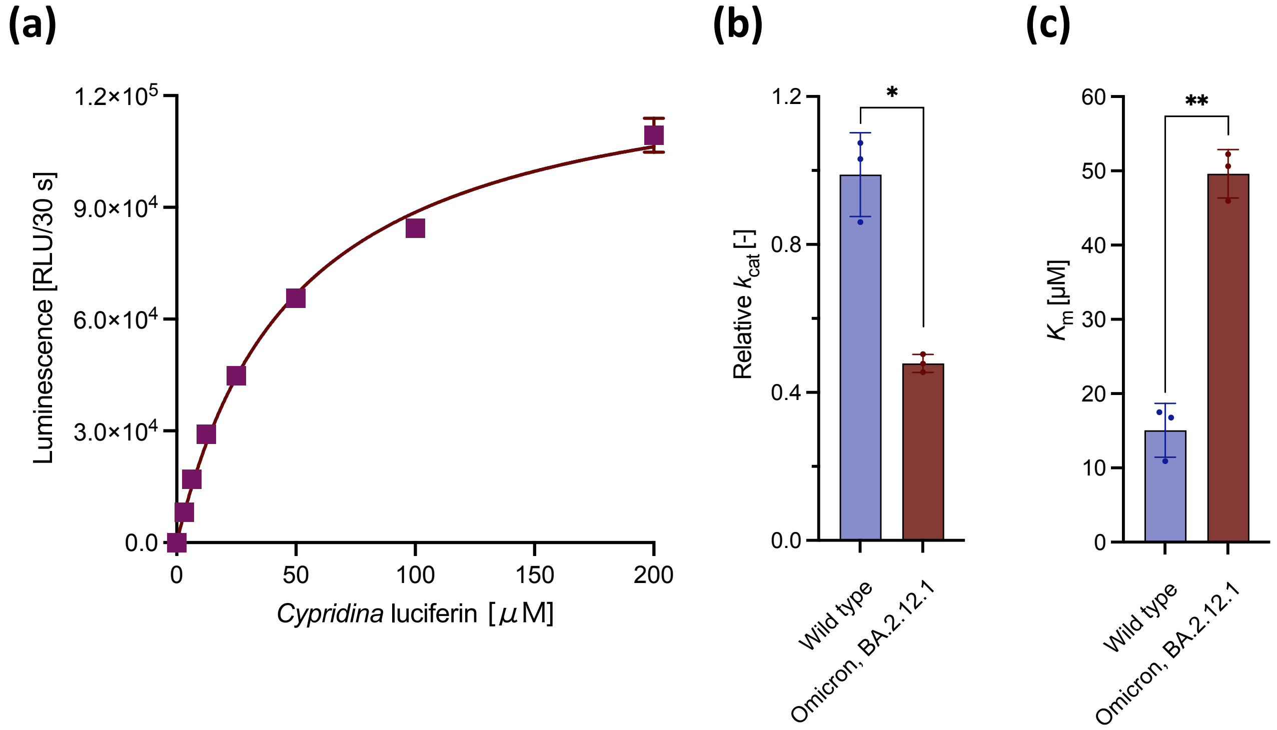

Figure S4. (a) Dose-dependent luminescence intensities: *Cypridina* luciferin (0-200 μM) in the presence of the trimeric protein of SARS-CoV-2 variant (omicron, BA2.12.1) (72 nM). (b) The relative k_{cat} value was calculated by normalizing the V_{max} value of the *Cypridina* luciferin/trimeric S protein (wild type) (72 nM) pair to 1.0. $^{*}P < 0.02$ (*t*-test). (c) K_m values of the trimeric S proteins for *Cypridina* luciferin. $^{**}P < 0.0001$ (*t*-test). Error bars represent the standard deviation of three independent measurements.

National Institute of Advanced Industrial Science and Technology (AIST)
1-1-1 Higashi, Tsukuba, Ibaraki 305-8566, Japan
Tel: +81-29-861-6158 / Fax: +81-29-861-6177
E-mail: r.nishihara@aist.go.jp / r.kurita@aist.go.jp

Table S4. V_{max} values of *Cypridina* luciferin with the trimeric S protein (BA.2.12.1)

	K_m [μM]	V_{max} [×10^5 RLU/30 s]
Trimeric S (BA.2.12.1)	49.6 ± 3.26	1.32 ± 0.06

Comment-4: For the diagnosis of an infection, it would be necessary detect the whole virus, not the isolated S proteins spiked in saliva. The authors should test real samples of infected patients or – if real samples are not available – they could by the inactivated virus and spice this into saliva. Then they will also see if the whole virus shows catalytic activity or if a lysis buffer is needed to disassemble the virus and release the S protein.

Reply-4:

As the reviewer pointed out, the test with real samples of infected patients will determine the practical utility of our developed BCL-based assay. The experiments using real samples of infected patients will be conducted in the next research project with the help of a hospital or an otherwise suitable institution equipped with a BSL-3 laboratory.

We would also like to thank the reviewer for the suggestion to experiment with inactivated viruses. However, we have decided not to carry out the suggested experiments due to the following reasons.

Firstly, S proteins of inactivated virus are known to differ in structure from those of active virus. Liu *et al.* have already reported that most S proteins on the inactivated virus adopt a post-fusion structure that differs from the pre-fusion structure of the S proteins on the active virus, which was determined by electron microscopy and tomography (for details, see: *Structure*, **2020**, *28*, 1218-1224.). Our study has targeted the recombinant S proteins that adopt a pre-fusion structure that is similar to that of the activated virus, not that of the post-fusion structure of the inactivated virus. Secondly, structural changes in the S protein may have significant impact on the enzymatic reaction between the S protein and the luciferin. As described in our **Reply 3**, the catalytic efficiency of the omicron-variant S protein is less than half of that of the wild type and its binding affinity for luciferin is more than three times lower (*cf.* Figure S4 and Table S4). Considering that the changes in the microenvironment around luciferin associated with amino-acid mutations (observed in omicron variant) have an impact on enzyme reactions, we cannot rule out an effect of the structural differences in S proteins between the inactivated and active virus on the enzymatic luminescence reaction. Therefore, in the phase of diagnostic development, we should test with the active virus, not inactivated virus. This is going to be conducted in a BSL-3 laboratory as described above.

We would like to emphasize once again that this is the first report to focus on a novel aspect of the isolated S protein's bioluminescent enzyme (luciferase)-like ability to catalyze the oxidative luminescence reaction of natural luciferin. We have also demonstrated the potential use of this pseudo-luciferase activity for a quantitative analysis of SARS-CoV-2 S protein in human saliva, albeit that the validation of the practical utility in real samples (using active whole viruses) is beyond the scope of this study.
